# Cellular immune endophenotypes separating early and late-onset myasthenia gravis

**DOI:** 10.1172/jci.insight.199679

**Published:** 2025-11-27

**Authors:** Jakob Theorell, Nicolas Ruffin, Andrew Fower, Chiara Sorini, Philip Ambrose, Valentina Damato, Lahiru Handunnetthi, Isabel Leite, Sarosh R. Irani, Susanna Brauner, Adam E. Handel, Fredrik Piehl

**Affiliations:** 1 Department of Medicine Huddinge, Karolinska Institutet, Stockholm, Sweden.; 2 Department of Neurology, Karolinska University Hospital, Stockholm, Sweden.; 3 Department of Clinical Neuroscience, Karolinska Institutet, and Center for Molecular Medicine, Karolinska University Hospital, Stockholm, Sweden.; 4 Nuffield Department of Clinical Neurosciences, University of Oxford, Oxford, United Kingdom.; 5 Department of Neuroscience and Psychology, University of Florence, Florence, Italy.; 6 Centre for Human Genetics, and; 7 Department Psychiatry, University of Oxford, Oxford, United Kingdom.; 8 Department of Neuroscience, Mayo Clinic Florida, Jacksonville, Florida, USA.

**Keywords:** Autoimmunity, Immunology, Neuroscience, Neuromuscular disease

## Abstract

The 2 main subgroups of autoimmune myasthenia gravis, a neuromuscular junction disorder associated with muscle weakness, are early- and late-onset forms, defined by onset before or after 50 years of age. Both carry acetylcholine-receptor autoantibodies but differ in sex ratios, genetics, and occurrence of disease-specific thymus inflammation. To distinguish the 2 forms by cellular immune phenotyping, we applied multimodal techniques, including deep spectral cytometric phenotyping and single-cell sequencing. Analysis of 2 independent cohorts identified immunological differences driven by 3 main lymphocyte populations. Lower frequencies of mucosa-associated invariant T cells and naive CD8^+^ T cells were observed in late-onset myasthenia, suggesting enhanced immune senescence. A highly differentiated, canonical NK cell population was reduced in early-onset myasthenia and negatively correlated with the degree of thymic hyperplasia. Using only the frequency of these 3 populations, correct myasthenia subgroup assignment could be predicted with 90% accuracy. These distinct immunocellular endophenotypes for early- and late-onset disease suggest differences in immunopathogenic processes. Along with demographic factors and other disease subgroup–specific features, the frequency of the identified cell subpopulations may improve clinical classification.

## Abstract 2

Patients with the autoimmune muscle disorder myastenia gravis get different treatments depending on if they are younger or older than 50 when diagnosed. We identify immune cell subsets that can better split the patients, so that biology rather than age alone, guides treatment choise.

## Introduction

Myasthenia gravis (MG) is an autoimmune neurological disease caused by autoantibodies targeting the neuromuscular junction, resulting in skeletal muscle weakness and sometimes life-threatening respiratory crises. A minor proportion of patients have only ocular symptoms, whereas the majority (80%–85%) have generalized MG involving multiple muscle groups(1, 2). Approximately 80% of patients have autoantibodies against the acetylcholine receptor (AChR), and the remaining carry autoantibodies targeting other neuromuscular junction proteins or are denoted as seronegative (3).

Patients with AChR autoantibodies (AChR-positive MG) can be subdivided into either early-onset or late-onset MG (EOMG and LOMG), based on disease onset before or after the age of 50 years, or the less common thymoma-associated MG with underlying paraneoplastic pathophysiology (4–6). EOMG and LOMG display contrasting features, implying at least partially different pathogenetic mechanisms: a) the EOMG group is more than 70% female versus less than 40% female in the LOMG group (4); b) carriers of HLA-B*08:01 are at risk of developing EOMG, whereas no HLA gene confers a marked risk specifically for the development of LOMG in the latest genome-wide association study meta-analysis (7); c) multiple autoantibody specificities may be observed in LOMG (e.g., anti-striated muscle antigens, the ryanodine receptor, type I IFNs, and/or IL-12) but not in EOMG (3, 8); d) inflammation of the thymus, hyperplasia, is common in EOMG. Such thymic hyperplasia is characterized by variable ectopic germinal center–like infiltrates with ongoing AChR autoantibody production, and surgical removal by thymectomy is clinically beneficial (8, 9). In contrast, since the thymus is normal for patient age (i.e., atrophied) in most cases of LOMG (10), its removal may be unnecessary. Because the biological age span of EOMG and LOMG can be expected to overlap, biological markers to distinguish the 2 forms potentially could serve to identify patients who stand to benefit from, for example, thymectomy (i.e., serving as an intermediate marker, or endophenotype, of the 2 subforms). However, prior studies exploring restricted immune cell populations, such as follicular helper T cell and memory T cell subsets, have thus far not identified distinct endophenotypes (11–16). The objective of this study was to explore whether immune cell populations differed in non-thymomatous AChR-positive MG of an age span overlapping between EOMG and LOMG. If so, this would strengthen the notion of relevant differences in the immunopathogenesis of EOMG and LOMG, as well as potentially help in directing patients to the correct treatments.

## Results

### Study cohorts.

To ensure the robustness of our findings, we studied 2 independent cohorts of patients with EOMG or LOMG (Table 1). The UK discovery cohort consisted of 28 patients with untreated AChR-positive MG, 12 patients with thymic hyperplasia-associated EOMG (sampled prior to thymectomy but categorized and selected for the study after the procedure), and 16 patients with LOMG. Samples from patients with ages close to 50 years were preferentially selected, and attempts were made to balance the sex ratio, to diminish the bias of the in-built age and sex differences between the patient subgroups on the results. In addition, matched thymic samples were available for 10 patients (9 EOMG and 1 LOMG). Age-matched samples from 20 healthy controls were included (Table 1). Deep spectral cytometry was performed, delineating B cells/CD4^+^ T cells/CD8^+^ T cells/γδ T cells, and NK cells/innate lymphoid cells (ILC), respectively (Supplemental Table 1; supplemental material available online with this article; https://doi.org/10.1172/jci.insight.199679DS1).

The Swedish (SE) validation cohort consisted of 8 patients with EOMG and 6 patients with LOMG whose data were collected between 2014 and 2020. All had symptomatic generalized MG at sampling with no ongoing immunomodulation, except 1 patient with LOMG who had received a course of intravenous immunoglobulin 4 weeks before sampling (Table 1). Five SE patients with EOMG had received thymectomy 2–20 years prior but experienced MG worsening at the time of sampling. Samples underwent a single-cell multiomics analysis, including CITE-Seq of 9 surface markers, whole transcriptome, and B and T cell receptor sequencing.

In addition, samples from 5 patients (3 UK and 2 SE), considered of uncertain MG subtype, were analyzed exploratively. These patients were either younger than 50 years of age but lacked thymic hyperplasia upon pathology examination, or 50 years of age and non-thymectomized. Furthermore, matched pre- and post-thymectomy PBMCs were available for 2 patients; these are shown in figures but not included in statistical analyses.

### Main cell subsets and supervised identification of discriminating populations.

**Figure 1:**
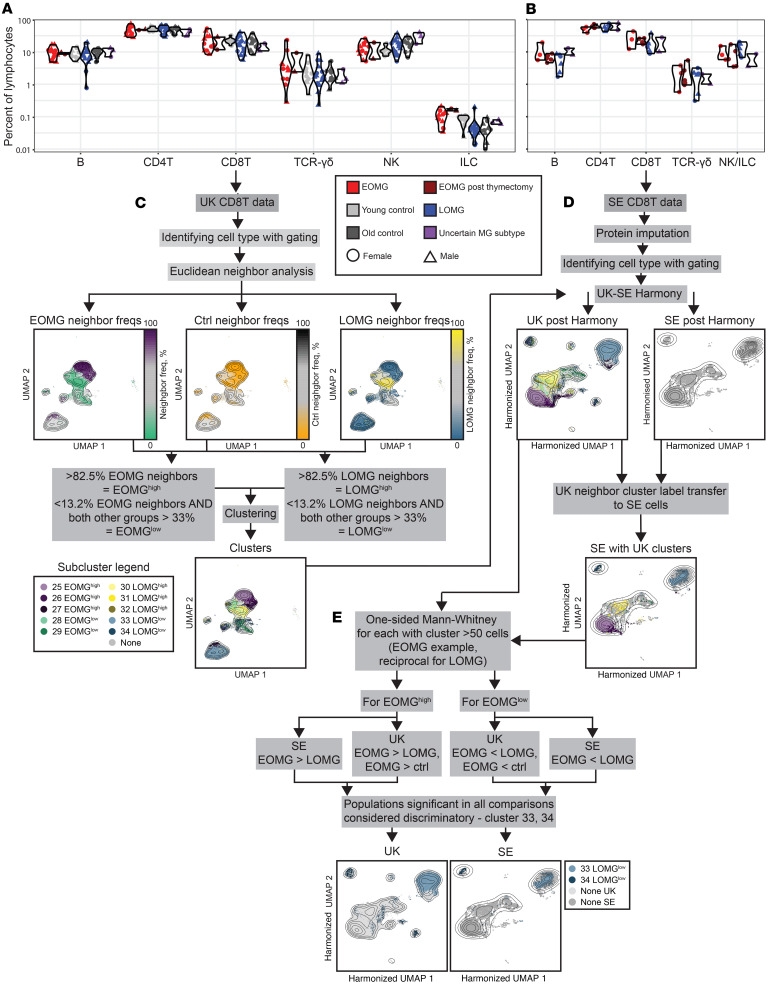
Overarching cell-type frequencies and overview of supervised clustering workflow. (**A** and **B**) Individual frequencies of the overarching cell types in the UK (**A**) and SE (**B**) cohorts. No differences reached significance after adjustment for multiple comparisons. (**C**–**E**) Supervised clustering workflow. In this case, CD8^+^ T cells are used as an example, but the workflow is identical for all cell types.

In order to find immunocellular endophenotypes discriminating the MG subsets, we first investigated differences in major cell populations in the 2 cohorts by a combination of conventional and Euclidean neighbor-enhanced gating (UK cohort, Supplemental Figures 1–3; SE cohort, Supplemental Figure 4). None of the major populations differed significantly, neither when comparing patient groups, nor with their age-matched controls (Figure 1, A and B). Next, the main cell populations were gated into subpopulations in the 2 cohorts (Supplemental Figure 1), revealing a differential abundance of some populations in the UK cohort (Supplemental Figure 5, A and B). After validation in the SE cohort, the remaining findings were lower percentages of naive as well as CD161^+^CD8^+^ T cells in patients with LOMG compared with patients with EOMG and age-matched controls (Supplemental Figure 5, A and C). However, the capacity to discriminate EOMG and LOMG at the level of individual patients was unsatisfactory (Supplemental Figure 5, D and E). To increase the discriminative capacity, we therefore designed and employed a supervised differential abundance-directed cell-selection method, related to previously published methods (17) but generalized to 3 groups. In total, 5% of ILC, 8% of CD4^+^ T cells and B cells, 10% of NK cells, 20% of CD8^+^ T cells, and 29% of γδ T cells from the UK cohort data were identified as potentially differentially abundant between the EOMG, LOMG, and control groups. These were further clustered into 61 subclusters (Figure 1C). Through neighbor-based cluster label transfer in a Euclidean space of common markers for the 2 cohorts, 11 of these candidate clusters were also identified in the SE cohort data in sufficient numbers (>50 cells) to allow for statistical inference (Figure 1D). Next, the frequency of these 11 remaining clusters was compared between the EOMG and LOMG groups, between the EOMG or LOMG groups and controls in the UK cohort, and between the EOMG and LOMG groups in the SE cohort. A cluster was selected if significantly different in all 3 comparisons (Figure 1E).

### Identification of 2 CD8^+^ T cell and 1 NK cell cluster as robustly differentially abundant.

**Figure 2:**
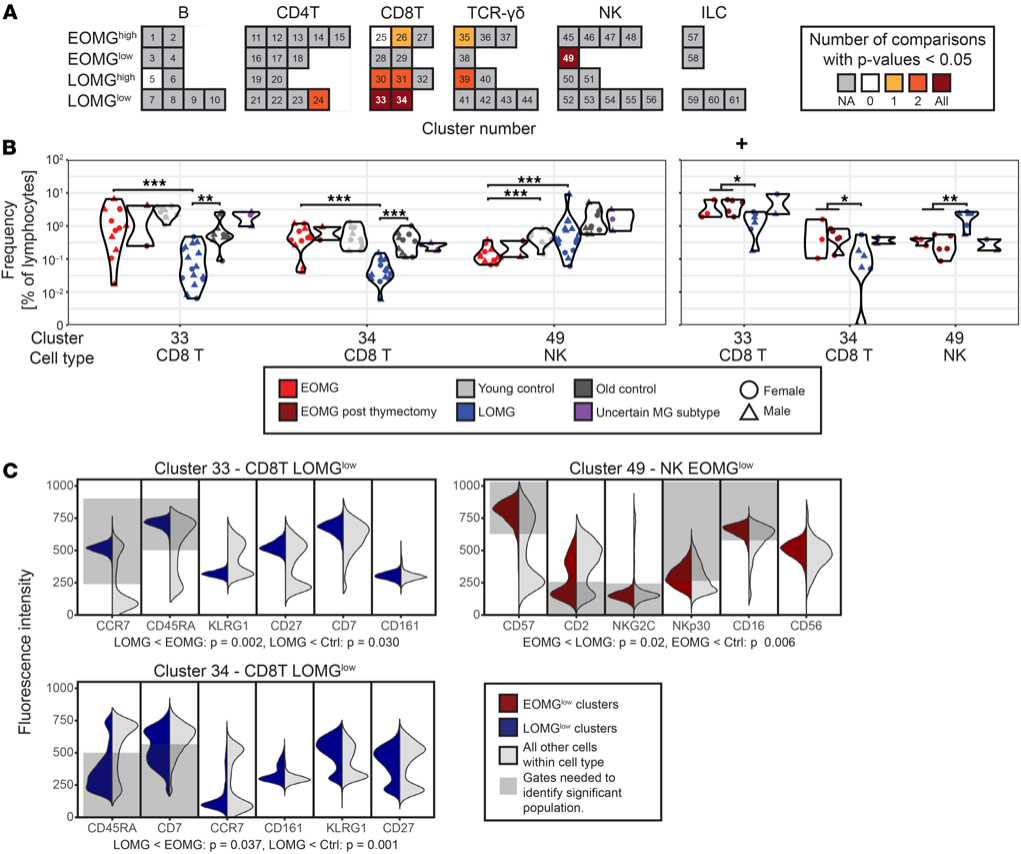
Identification and characterization of significantly different cell populations. (**A**) Overview of analyzed clusters and their respective significance in the iterative process. Dark red indicates significance in all 3 tests, which was the selection criterion for discriminatory clusters. Gray indicates that less than 50 cells were present in the SE data, so no comparisons were made. For all clusters where the significance was restricted to 1 or 2 populations, these came from the UK cohort. (**B**) Individual frequencies of the identified significantly different cell populations in the UK and SE cohorts. Mann-Whitney *U* tests were 1-sided for all comparisons, given that the cell populations were preidentified as over- or underrepresented in EOMG or LOMG compared with the other groups. *P = 0.05–0.005, **P = 0.005–0.0005, and ***P < 0.0005. As clusters have only been selected if all 3 independent comparisons had 1-tailed *P* values lower than 0.05, the false-positive error rate is 1/8,000, and with only 11 comparisons in total, no further adjustment for multiple comparisons was therefore included. (**C**) Selected surface markers defining the individual clusters in the UK dataset. The gray fields indicate gates that are sufficient to identify the significant cluster.

As expected for any method prone to overfitting, most clusters identified with the abundance-supervised method were significant only in 1 or 2 comparisons. However, 3 clusters (CD8T#33, CD8T#34, and NK#49) differed in all 3 comparisons (Figure 2, A and B, and Table 2). In the UK and SE cohorts, respectively, these clusters comprised 9% and 16% (CD8T#33) and 3% and 2% (CD8T#34) of all CD8^+^ T cells and 6% and 8% (NK#49) of total NK cells (Table 2). In line with the results of the initial manual gating analysis (Supplemental Figure 5), no distinct B cell or CD4^+^ T cell populations differed, whereas the frequency of the 2 CD8^+^ T cell populations was a discriminating factor between MG subsets and controls. Both CD8^+^ T clusters were smaller in the LOMG group (i.e., LOMG^lo^) as compared with the EOMG group and controls. These 2 CD8^+^ T cell populations could be distinguished with a few markers; the naive CD8T#33 cluster by CCR7 positivity alone, and CD8T#34 by being CD45RA^lo^CD7^lo^CD161^+^ (Figure 2C, Table 2, and Supplemental Figure 6).

In addition, a cluster of NK cells, NK#49, was reduced in frequency in the EOMG group compared with the LOMG group and age-matched controls (i.e., EOMG^lo^). This cluster displayed a canonical, highly differentiated NK cell phenotype and could be distinguished from other NK cells by a combination of CD16, CD57, NKp30, CD2, and NKC2C (Figure 2C, Table 2, and Supplemental Figure 6). Thus, based on a 3-way comparison in the UK cohort replicated in the SE cohorts, we uncovered 3 differentially abundant cell populations.

### Whole transcriptomic and T cell receptor profiling.

**Figure 3:**
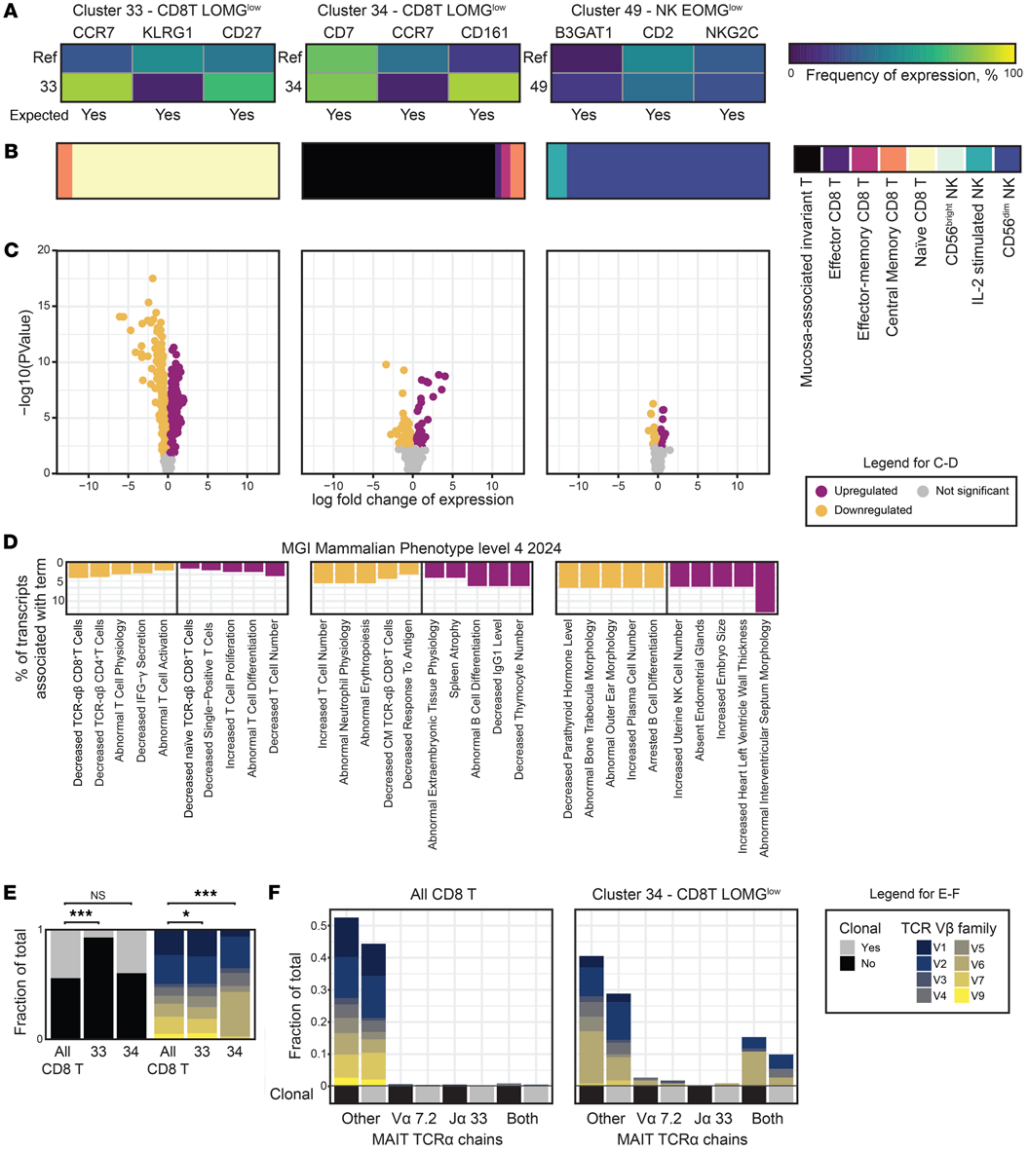
Characterization of significant cell populations. (**A**) Heatmaps indicating fraction of cells expressing genes among the top differentially expressed proteins for each of the 3 significant populations. Row 1 shows all cells apart from the population of interest and row 2 the population of interest. The 3 top proteins with a corresponding gene were selected for this analysis (the CD45RA isoform of CD45 not being useful in this context). (**B**) SingleR analysis identifying cluster 33 as naive CD8^+^ T cells, cluster 34 as MAIT cells, and cluster 49 as mature conventional NK cells. (**C**) Volcano plots indicating differentially expressed genes within the 3 cell subsets. (**D**) Top MGI mammalian phenotype terms associated with the top up- and downregulated genes for the respective cell populations. The length of the column is relative to the fraction of the total number of up- or downregulated genes for the cell subsets associated with the term. (**E**) Clonality and TCR Vβ chain family usage for the T cell populations. The first subfigure shows the distribution of clonality and TCR Vβ families for all CD8^+^ T cells, followed by columns for clusters 33 and 34. *P = 0.05–0.005, **P = 0.005–0.0005, and ***P < 0.0005. (**F**) Clonality and TCR Vβ split by MAIT TCRα chain usage. The first subfigure shows the distribution for all CD8^+^ T cells, whereas the second shows cluster 34. Color codes are the same as for **E**.

The SE cohort was used for in-depth transcriptomic characterization of the 3 identified cell populations. First, expression of the 3 top genes in each population validated a corresponding transcriptomic signal for the imputed surface proteins (Figure 3A). SingleR was applied to annotate known cell types (18), indicating that CD8T#33 comprised naive CD8^+^ T cells (93%), CD8T#34 mucosa-associated invariant T (MAIT) cells (87%), and NK#49 conventional CD56^dim^ NK cells (91%) (Figure 3B). To further characterize the latter, inhibitory killer Ig-like receptor (KIR) gene expression patterns were selectively analyzed, showing expression of KIR2DL1, KIR3DL1, KIR3DL2, and KIR2DL3, and low KIR2DL4 and KIR3DL3 expression, indicating a highly differentiated status (Supplemental Figure 7).

Next, compared with their corresponding cell types, differentially regulated genes for each of the clusters were identified (19): 401 upregulated, 370 downregulated for CD8T#33; 31 upregulated, 72 downregulated for CD8T#34; and 13 upregulated, 25 downregulated for NK#49 (Figure 3C and Supplemental Table 2). In a gene ontology analysis using EnrichR (20), cluster CD8T#33 was associated with T cell subset defects, CD8T#34 with broader hematopoietic defects, and NK#49 with B cell differentiation and decidual NK cells (Figure 3D and Table 2) (21).

As expected, T cell receptor (TCR) characteristics of the naive CD8T#33 cluster revealed a lower rate of clonality compared with the whole CD8^+^ T cell population (adjusted Fisher′s exact test *P* < 0.00001), whereas CD8T#34 did not (adjusted Fisher′s exact test *P* = 1) but displayed an increase of TCR-Vβ6 and reduction of TCR-Vβ7 (adjusted Fisher′s exact test *P* < 0.00001 in each case; Figure 3E and Table 2). Further, CD8T#34 TCR-α gene usage confirmed enrichment of MAIT cells (TCR-Vα7.2 and TCR-Jα33 in 26% of cells vs. 0.4% in the entire CD8^+^ T cell population; adjusted Fisher’s exact test *P* < 0.00001, Figure 3F and Table 2), with the most common prominent TCR-α phenotype being nonclonal MAIT cells expressing TCR-Vβ6 (11% of cells in CD8T#34 vs. 0.005% in the entire CD8^+^ T cell population; Fisher′s exact test *P* < 0.00001, Figure 3F and Table 2). In contrast, when comparing EOMG and LOMG in the SE cohort, the overall B cell receptor heavy gene mutation rates and TCR-beta gene V-family usage and clonality did not differ noticeably (data not shown).

### Relation to thymic pathology and capacity to discriminate MG subgroup membership.

**Figure 4:**
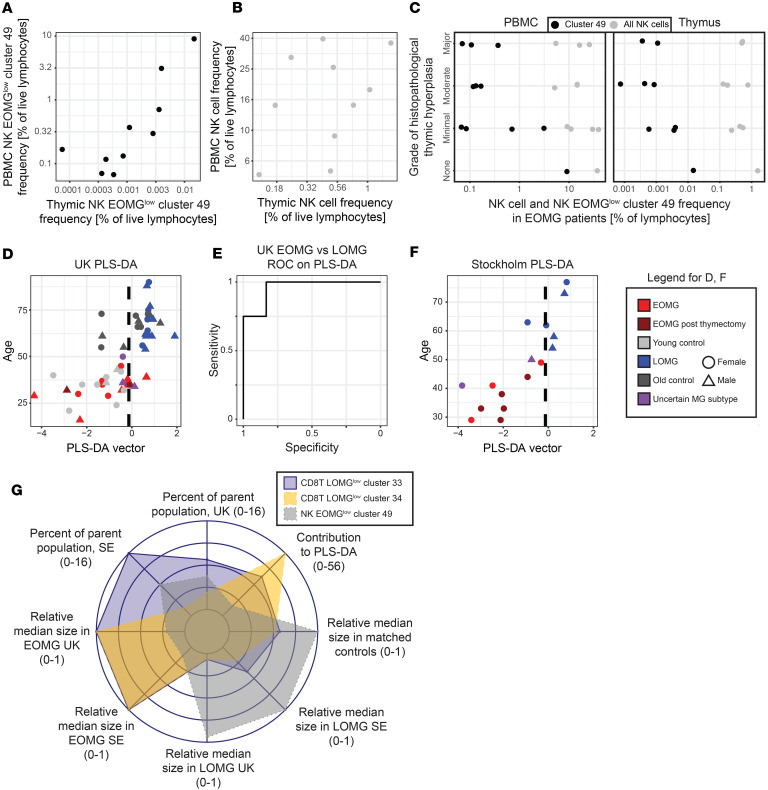
Thymic cell correlations and patient group discrimination. (**A** and **B**) Frequency of cluster 49 cells (**A**) and all other NK cells (**B**) in blood (*y* axes) as a function of the same cell population frequencies in thymus. Black dots indicate cluster 49 frequencies, whereas gray dots are the frequencies of NK cells after subtraction of cluster 49. (**C**) Frequency of cluster 49 in PBMCs and thymic cells from patients with EOMG in the UK cohort correlated to the grade of histopathological thymic hyperplasia. Colors have the same meaning as in **A** and **B**. (**D**) Partial least squares discriminant analysis based on the frequency of the 3 identified clusters, separating the patient populations in the UK cohort. Model generated only with the EOMG and LOMG myasthenia groups; post-thymectomy samples and control groups are merely displayed. (**E**) ROC curve based on the PLS-DA for the UK data. (**F**) The SE patient data displayed on the UK PLS-DA model, with the separation threshold inherited from the UK data. (**G**) Spider plot summing up some of the characteristics of the 3 identified discriminatory clusters. “Parent population” refers to all CD8^+^ T cells for clusters 33 and 34 and all NK cells for cluster 49.

Given the role of thymic hyperplasia in EOMG, we next investigated relationships between blood and thymic lymphocyte NK cells and the NK#49 cluster and thymic hyperplasia in a subcohort of 10 patients with paired samples available. There was a significant correlation between the NK#49 populations in the 2 compartments (Spearman’s ρ 0.83, *P* = 0.006, Figure 4A and Table 2), which, however, was not present for the total NK cell population (Spearman′s ρ 0.33, *P* = 0.34, Figure 4B). There was also a trend toward a negative correlation between the grade of hyperplasia and the logarithmic relative size of NK#49 in blood and thymic cell pools (Pearson′s correlation coefficients –0.59 and –0.66, respectively, adjusted *P* value 0.1 for both, Figure 4C and Table 2), which was considerably less pronounced for the entire NK cell population (Pearson′s correlation coefficients –0.42 and –0.41, adjusted *P* values 0.32 and 0.32, for logarithmic percentages of blood and thymic cells, respectively, Figure 4C). These findings point to a potential protective role for NK#49 in the control of thymic hyperplasia. Interestingly, the LOMG group showed a wide distribution of this subset, with partial overlap with the EOMG group.

Finally, we explored the capacity of the per-donor frequency of the differentially abundant clusters to correctly assign MG subgroup membership, using the UK cohort for training and the SE cohort for validation. Using partial least squares discriminant analysis, the optimally EOMG-LOMG separating vector based on the frequency of the 3 clusters was identified. This vector was influenced 35% by the frequency of cluster CD8T#33, 56% by CD8T#34, and 9% by NK#49 (Table 2 and Figure 4G). When it was used for classification of the UK cohort, 10 of 12 EOMG and 16 of 16 LOMG samples could be correctly classified (specificity of 100% and sensitivity of 83% for EOMG) (Figure 4, D and E). When the same vector and threshold was used for validation in the SE cohort, 8 of 8 EOMG and 5 of 6 LOMG samples were correctly classified (specificity 83%, sensitivity 100% for EOMG) (Figure 4F). Predicting whether a control individual was younger or older than 50 using the same vector and threshold was less successful (sensitivity 100% but specificity only 60% for young controls), indicating that the variance in the identified population frequencies is not only explained by age differences. Notably, 6 of 7 of the post-thymectomy EOMG samples and 2 of 3 and 2 of 2 of the uncertain cases in the UK and SE cohorts, respectively, were categorized as EOMG with this method. The single UK post-thymectomy EOMG sample that was wrongly categorized was slightly above the threshold and had moved 2% of the PLS-DA range from its pre-thymectomy value (Figure 4D). In summary, the identified cell populations (summarized in Figure 4G) differentiate EOMG and LOMG with reasonable sensitivity and specificity without consideration of any other distinguishing factors.

## Discussion

Leveraging 2 independent MG cohorts, spanning the traditional EOMG and LOMG age-based delineation cutoff at 50 years of age, we explored the potential existence of immune cell profiles that could distinguish these 2 major MG subgroups. Somewhat contrary to our expectations, major immune cell populations revealed only minor differences, where more sensitive supervised clustering only identified 3 divergent cell populations. This may partly reflect our conservative study design; a less conservative approach likely would have resulted in a larger number of divergent cell populations, while also increasing the risk of spurious findings. Nevertheless, the identified diverging cell clusters substantiate the notion of differences in underlying immune pathogenesis between EOMG and LOMG.

Underrepresentation of naive CD8^+^ T cells in LOMG aligns with immune senescence, known to increase risk of autoimmunity (22, 23). Notably, lower frequencies of MAIT cells in blood is linked to aging (24) and is also a feature associated with several autoimmune disorders (25). Thus, these cellular profiles of LOMG may reflect exaggerated immune aging compared with age-matched controls. A previous EOMG study found blood CD161^+^ T cells, which includes the MAIT population, to be lower in patients with EOMG compared with age-matched controls (16), but this observation could not be replicated here.

The size of the EOMG-distinguishing NK#49 cell cluster was strongly correlated in blood and thymic cell populations and showed a negative correlation trend in relation to thymic hyperplasia grades. NK cells have been proposed to play a regulatory role in autoimmune responses (26), and evidence of CD56^dim^ NK cell dysregulation in autoantibody-mediated neurological disorders, including MG, has been noted previously (27). Further, a recent study of expression quantitative trait locus mapping found that several instances of regulatory variation specific to NK cells were associated with risk of autoimmunity (28). It may therefore be asked whether there is a more complex background to the unusually strong HLA-B*08:01 linkage rather than only involvement of CD8^+^ T cell functions (29), especially since the HLA-B*08 haplotype has been shown to be associated with decreased NK cell functions and certain KIRs interact with HLA class I molecules (30, 31). Thymic NK cell frequencies in MG were captured in 2 previous studies (15, 32), but because subsets of NK cells were not described in any of the studies, results cannot be directly compared. This also holds for studies describing generally attenuated CD56^dim^ NK cells, with relative increases of CXCR5^+^ NK cells and decreases in NK cell cytotoxicity (33, 34). Of note, this population showed a wide frequency distribution among samples from patients with LOMG. Unfortunately, due to the lack of comparable disease activity scores from sampled individuals, we were not able to explore whether the size of the NK#49 cluster, either in patients with EOMG or LOMG, was also associated with disease activity, as has been shown for a noncanonical NK cell subset in a recent study (27). Other recent studies have investigated the blood and thymus lymphocyte compartments in MG, finding altered activated CD4^+^ T cell cytokine responses (15), increases of group 2 ILC and CD27^–^ TCR-γδ T cells (16), and changes in follicular helper T cells (12). No attempt to validate these findings was made in this study because no stimulation protocol was included, and follicular helper T cell frequencies could only be inferred indirectly based on expression in the SE dataset.

Pending validation by future studies, the identified cellular immune endophenotypes may have potential clinical relevance. For example, robust evidence for the efficacy of thymectomy for non-thymomatous MG derives from a single randomized controlled trial showing benefit for patients with MG aged 18–65 compared with prednisolone alone on 3-year disease severity outcomes (9) and with higher probability of minimal disease manifestations at 5 years (35). However, due to the composition of the study population, a beneficial effect in men, individuals aged 50 or older, and patients with mild disease remain unknown. Further, a retrospective Japanese study showed that only a minority of MG patients aged 50 or older undergoing thymectomy displayed hyperplasia (10). Still, these patients displayed less active disease at follow-up compared with patients without hyperplasia, which lends support to the notion that thymectomy is effective with thymic hyperplasia regardless of age. In the present study, a score based solely on the discriminatory cluster frequencies is presented, which separates patients with thymic hyperplasia with a 90% accuracy. Interestingly, 1 patient from each cohort, both patients exactly 50 years of age at the time of diagnosis, not offered thymectomy and therefore not classified as either EOMG or LOMG but uncertain MG subtype, clustered with the EOMG group. Even if this does not prove they would have benefited from thymectomy, it underscores that attempts to use case stratification based on biological features for directing patients to thymectomy are warranted.

Importantly, if a stratification score were to be constructed in a clinical setting, this immunophenotypic information would be integrated with other factors known to differ between EOMG and LOMG (e.g., levels of AChR antibody titers, sex, and, obviously, age). In addition, the 3 identified cell clusters shed light on underlying disease processes to be further explored in future studies. Our findings may also prove useful for characterizing immune phenotypes and tracking therapeutic responses, not least with potential future therapies relying on tolerogenic approaches (36).

There were several limitations to our study. First, cohorts were small, affecting our ability to identify discriminatory clusters and potentially also affecting external validity. In light of this, 3 precautions were taken. First, a high-resolution, supervised clustering method was employed, sensitively identifying cell clusters with endophenotypic discriminatory potential. Second, to limit overfitting, nearest neighbors of UK candidate cluster cells were sought in the SE cohort. In this way, 11 of the 61 clusters were identified in both cohorts. Third, a nested statistical test approach followed, identifying 3 of the 11 clusters as robustly discriminatory. Notably, when ranking the 61 clusters by size, the 11 clusters were interspersed among the largest 27 of the UK clusters (Supplemental Table 3). This underscores that a majority of the UK clusters were likely modeled from noise, but also indicates another study limitation, namely the relatively low number of collected cells per individual (*n* = 4,500) in the SE cohort. This likely explains why none of the 34 smaller UK clusters were reproduced in the SE cohort. Additionally, among the 3 discriminatory clusters, only 146 cells were present in the SE cohort MAIT cluster. These few cells were well-distributed over the EOMG donors, but this finding needs further external validation. On the other hand, in terms of robustness, cell populations that can be detected even with limited cell numbers have a higher utility in a clinical setting. Another limitation is that many EOMG samples were collected years before the controls in the UK cohort, although no discernible differences in cell viability relating to storage time could be seen. Further, 5 of 8 samples from patients with EOMG in the SE cohort were collected after thymectomy. However, all patients displayed relevant clinical worsening of generalized MG symptoms that prompted de novo institution of immunotherapy. Still, cell populations differing purely during the pre-thymectomy phase would have been excluded with our nested statistical approach. The lack of TCR sequencing for the UK cohort also meant that previously identified differences in TCRVβ chain family usage could not be reproduced (37) in the absence of a correlated surface phenotype. Finally, thymus pathology status was not available for most individuals with LOMG. This might have lowered the magnitude of difference between the groups, as some older individuals with thymic hyperplasia could have been present in the LOMG group. Finally, our work is wholly descriptive in nature, and further validation and mechanistic work is needed to address the discriminatory potential and usefulness of the identified cell subsets.

In conclusion, we identified a highly differentiated NK cell population being diminished in EOMG, negatively correlating with degree of hyperplasia, as well as 1 naive CD8^+^ T cell and 1 MAIT cell population that may reflect immune senescence in LOMG. If reproduced by others, these findings highlight the potential of immunological endophenotypes to rationalize treatment approaches, which is likely also relevant for future therapeutic strategies, such as efforts to develop tolerogenic therapies to suppress reactivity toward AChR epitopes.

## Methods

### Sex as a biological variable.

Given that the sex ratio is 2:1 female/male in EOMG and 1:1 in LOMG, male and female patients have been studied in this context. Two methods were used to circumvent the identified cell populations being associated with sex differences rather than MG subgroup. First, when selecting the cases for the UK cohort, a less sex-biased set of samples was chosen. Secondly, the healthy controls, especially for EOMG, were chosen with a more extreme sex bias than the cases (the controls are therefore not said to be matched for sex). This means that the UK EOMG samples were compared with the somewhat male-dominated LOMG group as well as the female-dominated young healthy controls. With this setup, a difference between the groups is unlikely to arise from sex alone.

### Study participants.

The UK discovery cohort consisted of 12 patients with EOMG, 16 patients with LOMG, 3 patients with uncertain MG subgroup membership (see Results for definition), and 20 age-matched controls. Of these patients, 8 patients with EOMG, 1 patient with LOMG, and 2 patients in the uncertain MG subgroup and all controls were recruited in Oxford. The Oxford patients were recruited between 1982 and 2002, whereas the controls were recruited between 2019 and 2023. The remaining 4 patients with EOMG, 15 patients with LOMG, and 1 patient in the uncertain group were recruited in Nottingham between 2014 and 2019. The SE cohort comprised 8 patients with EOMG, 6 patients with LOMG, and 2 patients with uncertain MG subtype recruited between 2014 and 2020.

### Procedures for cell freezing, batching, cytometry, and sorting.

PBMCs were separated within 6 hours of sampling and slowly frozen in media with 40%–90% FBS and 10% DMSO. Fresh thymic tissue was dispersed mechanically without enzymes, and cells were washed and frozen at 5°C per minute in 95% FBS and 5% DMSO. This was done by N. Willcox and colleagues in Oxford, in immediate conjunction with thymectomies.

For the UK cohort, all thawed samples were stained on the same day to avoid batch effects. The samples were thawed stepwise in successively warmer DNAse-containing medium in batches containing samples of all groups to introduce technical artifacts. All staining was performed in a staged manner to minimize the time differences in each staining step for the samples. A 5-step staining protocol was used for both panels, the details of which can be found at https://github.com/jtheorell/EO_vs_LOMG/tree/main/Data/Oxford/Documentation (commit b7eb797). Markers for both panels can be found in Supplemental Table 1. The data were manually acquired on a 5-laser Cytek Aurora instrument and the files saved raw. Despite a 40-year timeframe from the first to the last collected sample, no impact of storage time on the quality of cells could be discerned (Supplemental Figure 8).

For the SE cohort, 8 samples were stained and processed on each of 2 consecutive days. On each day, samples were thawed slowly in warm medium in a mixed order, so that every second sample was an EOMG sample. This was followed by staining with CITE-Seq antibodies (T/B/NK TotalSeq panel, BioLegend), including hashing antibodies specific to each sample, as well as with DAPI for dead cell detection. Markers for the CITE-Seq panel are in Supplemental Table 4. Sorting was conducted on a BD Biosciences Influx System instrument, selecting live singlet mononuclear leukocytes (Supplemental Figure 9). The whole sorting procedure took 38 and 31 minutes for the 2 experimental days, respectively. Details of the protocols and further sort documentation for the 2 experimental days can be found at https://github.com/jtheorell/EO_vs_LOMG/tree/main/Data/Stockholm/Sort_documentation

### Transcriptome, adaptive immune receptor repertoire, and surface marker (CITE-Seq) sequencing.

On each of the 2 experimental days, live sorted PBMCs were immediately brought to the Eukaryotic Single Cell Genomics Facility at SciLife Lab, Stockholm, Sweden. Here, the chromium 5′ version 3 kit (10x Genomics) was used, in combination with protocols for T cell and B cell receptor sequencing, as well as 9-marker CITE-Seq, including 8-sample hashing. This allowed for pooling of all samples from each experimental day, minimizing technical artifacts. Quality controls and further documentation about the processing can be found at https://github.com/jtheorell/EO_vs_LOMG/tree/main/Data/Stockholm/10X_quality_controls_and_documentation

### Handling of raw spectral flow cytometry standard files and gating for the UK cohort.

After export of the raw flow cytometry standard files from the Cytek Aurora instrument, files were unmixed using the flowSpecs package in R (38), including a manual curation step for the parameter names. After this, the files were imported into FlowJo (BD Biosciences), and the unmixing artifacts were corrected with a manual correction step, starting out with a square 0 matrix, which was filled with small corrective values upon visual identification of populations with spectral unmixing-related spurious correlations (similar to over- or under-compensation in conventional cytometry). After this step, gating was conducted in FlowJo in 2 ways: first, with a full conventional gating strategy as outlined in Supplemental Figures 1–3, and second, with a few gates excluding debris and dead cells and identifying the main cell populations (gates in Supplemental Figures 1, 2, and 4). These initial FlowJo-based analyses were followed by bioinformatic and statistical analyses in R, including further identification of the identified discriminant clusters through threshold filtering, akin to conventional gating.

### Bioinformatic analyses.

The full bioinformatic workflow (https://github.com/jtheorell/EO_vs_LOMG/tree/main/Scripts (commit b7eb797) includes a narrative description of the analyses in the Supplemental Methods.

### Statistics.

For all analyses concerning differences between groups, Wilcoxon rank-sum tests were used. Samples with an uncertain MG subtype or thymus populations and UK post-thymectomy PBMC samples were not included unless explicitly stated. Notably, for the SE cohort, PBMCs obtained after thymectomy were included in the statistical analyses. Use of other statistical tests has been specified in the text (e.g., Pearson’s correlation, Spearman′s rank correlation coefficient, and Fisher′s exact test). The reason for the use of Pearson’s correlation for tests involving the ordinal thymic hyperplasia scores is that rank correlations perform poorly when multiple values are identical.

For the nested supervised clustering method aimed at identifying discriminatory clusters, statistical difference was defined by a 1-tailed *P* value less than 0.05 for all 3 comparisons (UK EOMG vs. LOMG and EOMG/LOMG vs. control, and SE EOMG vs. LOMG). The direction of the tail was defined by the population size in the patient group of interest compared with the other patient group and the controls. In other words, a population selected due to its smaller size in EOMG was only statistically tested to be smaller in EOMG compared with the other groups in both cohorts. This setup with 3 independent tests lowered the false-positive error rate to 0.05^3^ = 0.000125 or 1 in 8,000 tests. If we had not used this nested approach, only making 11 comparisons in total, and used the strictest correction for multiple comparisons (Bonferroni’s correction) at a *P* value threshold of 0.05, the corrected *P* value would have been 0.05/11 = 0.0045 or 1 false-positive in 220 tests, which was considered too high a risk with the dataset sizes in question. Had we instead combined the nested approach with further testing for multiple comparisons, the result would have been 0.000125/11 or 1 false-positive in 88,000 tests, which on the contrary would be too conservative. Thus, our current strategy contains a strict *P* value selection threshold, and no additional adjustment for multiple comparisons was made for the nested identification of discriminatory clusters. A false-detection rate procedure was applied for all other multiple comparisons.

### Study approval.

All individuals recruited in Oxford gave oral consent (keeping only information about their age and sex), as approved by the Central Oxford Research Ethics Committee (COREC 1702). The Nottingham patients provided written consent, as approved by the Yorkshire and Humber Research Ethics Committee (REC16/YH/0013). The Swedish patients provided written consent, as approved by the Swedish Ethical Review Authority (dnr 2009/2107-31/2, last amended 2024-07377-02, and dnr 2016/827-31, last amended 2024-02236-02).

### Data availability.

Values for all data points in graphs are reported in the Supporting Data Values file. To reproduce the full analysis workflow and access all the preprocessed data, all the underlying data for the whole analysis workflow, including the raw count matrices and all data resulting from the initial spectral cytometry analyses, can be accessed at https://github.com/jtheorell/EO_vs_LOMG/tree/main/Data/Stockholm/Raw_data (commit b7eb797) where the SE dataset is split into 3 files due to size restrictions. The Kotliarov dataset is also available here (39).

All unmixed UK cohort flow cytometry standard files can be directly downloaded (http://flowrepository.org/id/FR-FCM-Z86X). Raw fastq files from the SE cohort can be viewed at https://doi.org/10.48723/9gg8-ry45 Only researchers who have an ethical permit can apply for access to these files, given European personal data protection laws.

## Author contributions

JT was responsible for conceptualization, methodology, software, formal analysis, investigation, data curation, writing (original draft, review, and editing), visualization, and project administration; NR performed validation and investigation and reviewed and edited the manuscript; AF, IL, LH, and PA provided resources and reviewed and edited the manuscript; CS performed formal analysis and visualization and reviewed and edited the manuscript; VD reviewed and edited the manuscript; SRI performed validation, provided resources, reviewed and edited the manuscript, and supervised the study; SB and AEH performed validation, provided resources, curated data, and reviewed and edited the manuscript; and FP conceptualized the study, provided resources, curated data, supervised the study, acquired funding, and assisted with writing the manuscript (original draft, review, and editing).

## Funding support

National Genomics Infrastructure, Stockholm, Sweden, funded by Science for Life Laboratory and the Knut and Alice Wallenberg Foundation.

- EJP-RD Joint Translational Program 2023 (OptiMyG, grant 2023-00533).
- Swedish Brain Fun
- Stockholm Region (grant FoUI-987565).
- Swedish Research Council (grant 2023-00533).
- A senior clinical fellowship from the Kogod Centre on Aging, Medical Research Council (MR/V007173/1).
- A Wellcome Trust Fellowship (104079/Z/14/Z).
- National Institute for Health Research (NIHR) Oxford Biomedical Research Centre.
- MyAware.
- MRC funding (MR/X022013/1).

## Supplementary Material

Supplemental data

Supplemental tables 1-4

Supporting data values

## Figures and Tables

**Table 1:**
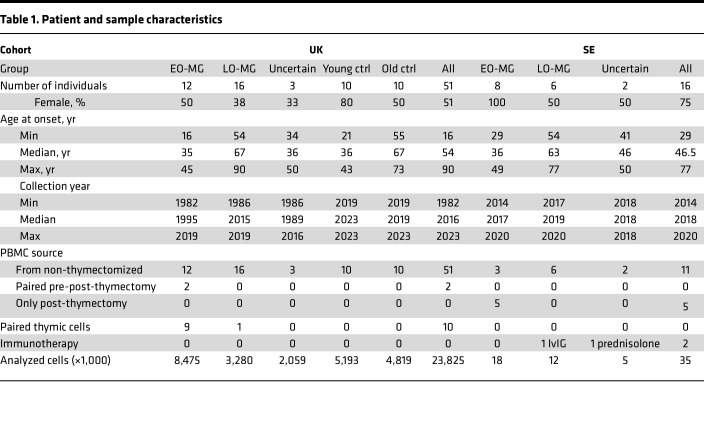
Patient and sample characteristics

**Table 2:**
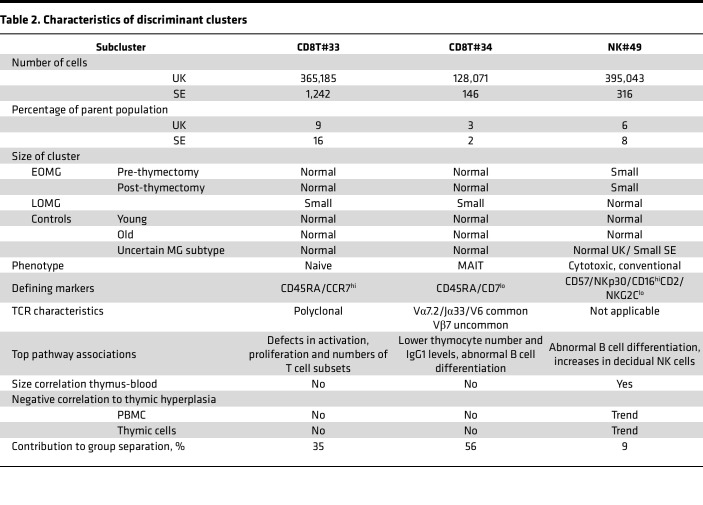
Characteristics of discriminant clusters

## Version 1 11/27/2025

In-Press Preview

## Version 2 01/09/2026

Electronic publication
