# Supplementary material for: Cellular immune endophenotypes separating early and late-onset myasthenia gravis
Source: JCI Insight. 2025 Nov 27;11(1):e199679. doi: 10.1172/jci.insight.199679 (PMC12890496; doi:10.1172/jci.insight.199679)
Supplement: Supplemental data [file jciinsight-11-199679-s198.pdf]

Supplementary methods

Bioinformatic analyses

Annotated code for all the processes described below is found at https://github.com/jtheorell/EO_vs_LOMG/tree/main/Scripts. The processing for both cohorts has been conducted in R (1) with a few exceptions, then spelled out below. "Packages" referred to below are subprograms under the R umbrella.

Euclidean neighbor-enhanced gating

For separation of NK cells and ILC in the UK cohort for the Euclidean neighbor clustering analysis, as well as for all separation of all cell populations in the SE cohort for the same analysis, a variant of conventional gating was used. Here, the clearly positive and negative populations for each relevant marker was first defined, e.g. CD3 clearly negative, CD19 clearly positive were considered B cells. The cell type for borderline populations were however initially not defined. After this procedure had been conducted for all relevant cell types, a label transfer procedure was conducted for all individual cells in the areas with undetermined cells. First, the nearest neighbor was identified on a per-cell basis. In the case of ILC/NK in the UK data, the Euclidean nearest neighbor space was made of NKp30, HLADR, CD183, CD161, CD16, CD34, CD56, CD8, CD127, CD27, CD57, NKG2A, CD2, NKG2C, CRTH2, CD7, CD117, CD218a. In the case of the SE data, the nearest neighbor space was instead made up of the measured surface markers CD3, CD4, CD8, CD11c, CD14, CD16, CD19, CD45 and CD56 together with the full transcriptome, integrated to a 20-dimensional latent space identified with totalVI (2).

In the case of the UK data, the nearest neighbor cloud was reduced to a high number (tens to thousands) of per-cell type subclusters prior to the nearest neighbor analysis, to limit computational needs. In the case of the SE data on the other hand, the actual nearest neighboring cell was used. The cell type label from this nearest neighbor was then transferred to each of the undetermined cells. With this procedure, all cells in the datasets were defined and the risk of erroneous labeling due to stochastic variation in signal intensity in the border regions between clearly positive and clearly negative populations.

UK Cohort - Euclidean neighbor smoothing analysis

After exclusion of debris and dead cells, the cell populations of interest were identified by gating using FlowJo software v 10.8-10 (BD Life Sciences). The unmixed and transformed fluorescence data for each cell was then exported to a separate .csv file for each donor and cell type (B, CD4T, CD8T, $\gamma\delta$-T and ILC/NK, Supplementary figure 4-5). In addition, unmixed and transformed fluorescence data for a random subset of live lymphocytes from each donor and panel was exported as separate file and further, information about the total number of live lymphocytes was exported per donor. This information was then imported into R and integrated to objects on a per cell type basis, together with per-sample metadata. ILC and NK cells were imported together into R and separated with the Euclidean neighbor-enhanced gating approach described above (Supplementary Figure 6). A few minor adjustments to the ILC population were made after the Euclidean neighbor step: a small

population of NKG2A$^+$CD127$^+$ cells were defined as NK cells, and two small groups of cells, CD127+CD8+, determined to be CD8 T cells with low surface CD3 expression, and CD127$^-$CD117$^+$ progenitor cells, respectively, were excluded.

It is worth noting that CD4/CD8 double positive and double negative T cells were also initially analysed, but as these populations were so small, especially in the SE cohort, it was not possible to draw any statistical conclusions based on their result, and they were therefore excluded from further analyses.

Following this the identification of cells with an over- or underrepresentation in EOMG or LOMG compared to the other category as well as controls were identified on a per-cell type basis. Here follows a description of this procedure for CD4 T-cells, but the procedure was the same for all. First, the peripheral blood mononuclear cell (PBMC) samples collected before thymectomy from EOMG patients, LOMG patients and controls were identified for neighbor selection. In other words, samples of unclear myasthenia subtype, as well as EOMG samples post-thymectomy and thymus samples were never used as neighbors in the further analysis.

After this, the number of cells to be used as neighbors was identified for each sample. This was done in a convoluted fashion, to ensure equal representation of each donor and group. Each donors number was related to the total number of lymphocytes from that individual, so that the neighbors for each donor within a group would be represented by the same number of total lymphocytes from that donor, and each group would be represented in the same way by the same number of lymphocytes. Exemplifying this with a seven-donor, three group example for CD4 T cells:

Group	EOMG	EOMG	EOMG	LOMG	LOMG	Ctrl	Ctrl
Donor	1	2	3	4	5	6	7
Lymphocyte number	100000	200000	150000	80000	230000	125000	200000
CD4T number	50000	120000	100000	55000	170000	100000	120000
CD4T fraction	0.5	0.6	0.66	0.69	0.74	0.8	0.6
Lowest lymphocyte number per group	100000	100000	100000	80000	80000	125000	125000
Number of lymphocytes per group with highest equal number per donor	300000	300000	300000	160000	160000	250000	250000
Highest possible lymphocyte number with equal group size	160000	160000	160000	160000	160000	160000	160000
Number of lymphocytes per donor with equal donor and group size	53300	53300	53300	80000	80000	80000	80000
Number of neighbor CD4T cells, related to fraction of lymphocytes	26650	31980	35178	55200	59200	64000	48000

If, like in the example, the neighbor set exceeded 100000 total cells, the individual cell number were adjusted down to make up a total of 100000, keeping to the same donor:donor ratios as before the downsampling.

Following this, a random subset of cells was selected as neighbors, with the individual number specified by the above calculation. Then, for all cells in the dataset, the closest x neighbors (see below) were identified among this neighbor set, and the fraction of these neighbors belonging to the EOMG, LOMG and control groups was calculated. Following this, a new subset of the dataset was used as neighbors, and the average frequency of neighbors between this round and the previous round was calculated. This bootstrapping procedure was repeated for the whole dataset so many times, that the addition of the new set of values added less than 1% to the total squared variance of the repeat and all the previous repeats. In reality, this meant that the boostrapping procedures were repeated between 15 and 20 times. Furthermore, the whole procedure was repeated with 5, 15 and 45 neighbors. In total therefore, each event was exposed to at least 5x15 + 15x15 + 45x15 neighbors, producing three sets of frequency values, one for each number of neighbors. This procedure utilized the DepecheR R package (3).

When the average fraction of neighbors had been calculated per number of neighbors, individual cells of interest were identified as such that either had neighbors with more than 82.5% neighbors (2.5 times the expected 33%) from EOMG or LOMG, or where one of the patient groups were represented by less than 13.2% (the expected 33% divided by 2.5) of the neighbors and both the other patient group and the controls were represented by more than the expected 33% of the surrounding events, this last criterion included to avoid doubly identifying evens as high in EOMG and low in LOMG, e.g. This led to the identification of $EOMG^{high}$, $EOMG^{low}$, $LOMG^{high}$ and $LOMG^{low}$ cells for each cell type. As a final filter, only cells that were classified as belonging to one of the $EOMG^{high}$, $EOMG^{low}$, $LOMG^{high}$ and $LOMG^{low}$ populations with all three sets of neighbors, were defined as potentially discriminatory single cells. These cells were then clustered together, per cell type, into the four categories $EOMG^{high}$, $EOMG^{low}$, $LOMG^{high}$ and $LOMG^{low.}$

To clarify if these cell categories should be further subclustered, Bayesian Information Criteria (BIC) were calculated, using the mclust package (4) for each of the $EOMG^{high}$, etc, subsets of cells for each cell type with more than 5000 cells. All subsets with an improved BIC criterion of more than 5% for any of the included cluster models were subsequently subclustered using the DEPECHE algorithm (cite DepecheR). As all clusters fulfilled this criterion, it was in the end only the number of cells that determined if the cluster was subclustered or not, and only ILC clusters were smaller than this number. One exception was made to this: it was subsequently clarified by statistical analyses that all three NK cell clusters belonging to the $EOMG^{low}$ set were both phenotypically very closely related (differing in CD8 and CD2 expression only) and were all identified individually as significant in all three independent statistical analyses, both for the UK and SE cohorts, so the $EOMG^{low}$ NK cells were in subsequent iterations of analyses collapsed to one cluster. This clustering-subclustering approach resulted in a final 61 clusters for the UK cohort.

SE cohort - preprocessing

For cell surface antibody/CITE-seq sequencing data as well as BCR/TCR sequencing data, cellranger 6.1.2 (10X Genomics, Pleasanton, California, USA) was used. For gene expression data, cellranger 7.0.1 was used, including for filtering of empty droplets. For the BCR/TCR data, only the filtered

contigs were kept from cellranger, and all downstream analyses were conducted with the changeo-10X (5) functions from the Immcantation suite, using the Docker (6) mirror. In the case of BCRs and TCRs, clone analysis was performed on the heavy chains and TCR-beta chains only, respectively.

After these stages, the data was imported into R and cells were excluded because of low library size, low numbers of features and high percentages of mitochondrial genes. This, and all other generic preprocessing steps utilized the OSCA package suite (7). Following this exclusion, the cell surface data was integrated and the hashing antibodies were used to separate the donors, using the dropletUtils package (8). This pre-processing was done for each of the six total runs (three per day) and subsequently, the data was combined into one file with a total of 35734 cells.

Kotilarov control dataset preprocessing

The dataset, including both gene expression and surface protein data was imported through the scRNAseq package (9). Exclusions based on small library size, low number of features and high percentage of mitochondrial genes were conducted separately for the two experimental days that the data was generated on.

SE and Kotliarov dataset merging and totalVI

After these separate preprocessing steps, 20182 common transcripts were identified, and the transcriptomic data was merged and exported. After this, the complete Kotliarov protein data was exported as a .fcs file, and investigated in FlowJo (Becton Dickinson, Franklin Lakes, New Jersey, USA). This file, and the associated processing can be found at https://github.com/jtheorell/Cite_seq_FCS. In this processing, the following markers were excluded as they were not or very lowly or expressed: CD14, CD133 (stem cell marker expressed in B cells here, without correlation to transcript in this or other datasets), CD134, CD138, CD152, CD183, CD184, CD197, CD206, CD223, CD273, CD275, CD294, CD357, CD366, IgA, IgM, TCRgd (low expression and low correlation to CD3). Annexin V is also excluded, as the dataset seemingly has undergone previous pre-processing for exclusion of dead cells. This left a total of 62 markers:
BTLA, CD1c, CD1d, CD2, CD3, CD4, CD5, CD7, CD8, CD10, CD11b, CD11c, CD13, CD16, CD18, CD19, CD20, CD21, CD24, CD25, CD27, CD28, CD31, CD32, CD33, CD34, CD38, CD39, CD40, CD45RA, CD45RO, CD56, CD57, CD62L, CD64, CD69, CD70, CD71, CD80, CD86, CD90, CD103, CD117, CD123, CD127, CD141, CD161, CD163, CD185, CD194, CD195, CD196, CD244, CD278, CD279, CD303, CD314, CX3CR1, HLA-ABC, HLA-DR, IgD and KLRG1. This dataset was exported. A further subset, containing only surface markers overlapping with the SE data was further exported: CD3, CD4, CD8, CD11c, CD16, CD19, CD56.

After this, two procedures utilising totalVI (2) were used. In the first instance, only the count matrices for the gene expression and the reduced set of proteins included in both datasets were used, and a normalised level of these proteins was computed for each cell based on both the full single-cell transcriptomic and protein expression. In the second instance, the dataset including all proteins from the Kotliarov dataset was used together with the transcriptomic data to impute the expression of the

proteins in the SE dataset that were not measured originally. The data from the normalised and imputed proteins was then combined.

Reduction of spurious protein-protein correlations

After the totalVI analysis, it was evident that new artifactual correlations between proteins, hitherto unseen in the Kotliarov data, had appeared. To abate this, a linear modeling approach was created. First, the data was clustered using a rough clustering algorithm (the clusterCells function in the OSCA scran package), creating clusters with sizes varying from 38 to 8603 cells, focusing on the Kotliarov dataset only. Then, a random subset of 38 cells was retrieved from each cluster, thus in reality downsampling all the data maximally, to increase diversity of the dataset. With this diverse dataset, a linear model was created, identifying the "correction matrix" that minimised the differences between the original and imputed markers. This correction matrix was then applied to the SE cohort dataset. An example of the effects can be viewed in supplementary figure 10.

Euclidean gating and SingleR definitions of cell types

For the SE data, the Euclidean gating concept, described above, was expanded somewhat, to allow for the analysis of gamma-delta T cells. First clearly definable B cells, NK cells, myeloid cells, doublets as well as bulk T cells were defined. After this, T-cells lacking an alpha-beta-TCR, as measured separately, were classified using Singler using labels defined in the Monaco dataset (10). Cells with a high probability of being gamma-delta T cells, defined as the second most likely candidate cell type being less than 10% as likely as gamma-delta T cells were then defined as such. After this procedure, clear CD4 and CD8 T cells (as well as Double-positive and double-negative T cells, which were excluded after the procedures were finished due to their minor size) were defined among the remaining T cells. This was followed by categorisation of the remaining non-determined cells into one of the cell types delineated above using the Euclidean gating concept. Finally, a second round of SingleR classifications were performed for the small group of CD4 and CD8 T cells lacking a functional alfa-beta-TCR, and cells within this category that were classified as gamma-delta T cells were moved into this category (summary in supplementary figure 7).

Joint cohort analyses: Harmony-based integration and cluster label transfer

Next, the protein data from the UK cohort and the imputed data from the SE cohort were integrated. This was performed on a per-cell type basis. First, a common set of markers between the UK and SE data and relevant to the cell type in question were defined:

Celltype	B	CD4 T	CD8 T	TCR-$\gamma\delta$	NK	ILC
Markers	CD38<br>CCR7<br>CD95<br>CD45<br>CCR4<br>IgD<br>CD11b<br>CD27<br>CD20	CD38<br>KLRG1<br>CCR7<br>CD95<br>CD25 CD4<br>CCR4<br>CD71<br>CD11b<br>CD27	CD38<br>KLRG1<br>CCR7<br>CD95<br>CD25<br>CCR4<br>CD71 CD8<br>CD11b<br>CD27	CD38<br>KLRG1<br>CCR7<br>CD95<br>CD25<br>CCR4<br>CD71<br>CD8<br>CD11b	NKp30<br>HLA-DR<br>CCR7<br>CD183<br>CD161<br>CD16<br>CD34<br>CD56	NKp30<br>HLADR<br>CCR7<br>CD183<br>CD161<br>CD56<br>CD127<br>CD27<br>NKG2A

	CD45RA<br>CD31<br>CD24<br>IgM	CD64<br>CD20<br>CD45RA<br>CD2 CD31<br>CD161<br>CD7<br>CD24<br>CD152	CD64<br>CD20<br>CD45RA<br>CD2 CD31<br>CD161<br>CD7<br>CD24<br>CD152	CD27<br>CD64<br>CD20<br>CD45RA<br>CD2 CD31<br>CD161<br>CD7<br>CD24<br>CD152	CD8<br>CD127<br>CD27<br>CD57<br>NKG2A<br>CD2<br>NKG2C<br>CRTH2<br>CD7<br>CD117<br>CD218a	CD2<br>CRTH2<br>CD7<br>CD117<br>CD218a

With the relevant set of markers, a cell-type specific 10-dimensional PCA was constructed for the UK and SE data separately. This data was then integrated to a common 10-dimensional PCA using the Harmony package (11). In this integrated 10-dimensional space, the nearest neighbor of each SE cell was sought after in the UK data, and the cluster label was transferred from the UK to the SE neighbor. The resulting common clusters between the UK and SE cohorts were then statistically investigated and only clusters with significantly differences with the same direction in both cohorts and between patients and controls in the UK cohort were considered discriminatory clusters, see main manuscript.

Singler annotation of known cell types for discriminatory clusters

In this analysis, two different annotation libraries were used, as they had different resolution for the CD8T and NK cell compartment; for the CD8 T cell discriminatory clusters, the Monaco dataset (10) and for the NK cell cluster, the Human cell atlas (12). In both cases, a reduced set of labels were allowed: for the CD8 T cell clusters, "Naive CD8 T cells", "Central memory CD8 T cells", "Effector memory CD8 T cells", "Terminal effector CD8 T cells" and "MAIT cells". For the NK cells, the labels were: "NK_cell", "NK_cell:IL2" and "NK_cell:CD56hiCD62L+".

EdgeR and EnrichR analyses on discriminatory clusters as well as random cluster counterparts

In this analysis, the standard pipeline illustrated for single-cell RNA seq data in the OSCA book (7), utilizing pseudo-bulking and EdgeR (13) was used to identify genes either up- or downregulated by the cells in the three discriminatory clusters. Selection of the significant markers was done with the topTags function, with an adjusted p-value of 0.05.

To further interpret the functional implication of the identified sets of genes, EnrichR (14) was used, through its R interface, primarily with the mouse genome informatics (MGI) mammalian phenotype term library (15), due to the high relevance for the subject of the MGI terms, but also with the ARCHS4 transcription factor coexpression database. To avoid identifying terms/transcription factors associated to the whole cell type rather than the discriminatory cluster, 100 random sets of cells with the same size as the original discriminatory clusters and drawn from the overarching cluster cell type (CD8 T or NK cells), were created. These were then analysed with EdgeR and EnrichR, and terms/transcription factors associated to more than 5% of these random clusters were filtered out from the results for the discriminatory clusters.

TCR and BCR analyses

All analyses were performed within the Immcantation framework.

BCR clones were defined with the Changeo DefineClones function using Hamming distances. The cutoff was manually set upon inspection of the distance distribution for the dataset. The within-clone distances were confirmed to be in the 0-4 Levenshtein distance range with less than 4% of distances above 0. The light chains were distributed over the 0-14 Levenshtein distance range with less than 3% of distances above 0. Mutation rates were calculated with the "observedMutations" function in the Shazam package.

TCR clones were also defined with the Changeo DefineClones function using Hamming distances. The cutoff was manually set upon inspection of the distance distribution for the dataset. After clustering, the within-clone distances were confirmed to be in the 0-1 Levenshtein distance range with less than 0.7% of distances above 0.

Partial least squares discriminant analysis

This analysis was performed with the mixOmics R package (16). The percentage of the contribution from each cluster variance to the PLS-DA vector was identified by squaring the X loadings for the respective markers.

Bibliography

1. R Core Team, R: A Language and Environment for Statistical Computing. (2017). Deposited 2017.
2. Joint probabilistic modeling of single-cell multi-omic data with totalVI - PubMed. Available at: https://pubmed-ncbi-nlm-nih-gov.proxy.kib.ki.se/33589839/ [Accessed 21 November 2024].
3. Determination of essential phenotypic elements of clusters in high-dimensional entities-DEPECHE - PubMed. Available at: https://pubmed.ncbi.nlm.nih.gov/30845234/ [Accessed 9 September 2022].
4. L. Scrucca, C. Fraley, T. B. Murphy, A. E. Raftery, *Model-Based Clustering, Classification, and Density Estimation Using mclust in R* (Chapman and Hall/CRC, 2023).
5. N. T. Gupta, *et al.*, Change-O: a toolkit for analyzing large-scale B cell immunoglobulin repertoire sequencing data. *Bioinformatics* **31**, 3356–3358 (2015).
6. D. Merkel, Docker: lightweight linux containers for consistent development and deployment. *Linux journal* **2014**, 2 (2014).
7. R. A. Amezquita, *et al.*, Orchestrating single-cell analysis with Bioconductor. *Nat Methods* **17**, 137–145 (2020).
8. Detection and removal of barcode swapping in single-cell RNA-seq data | Nature Communications. Available at: https://www-nature-com.proxy.kib.ki.se/articles/s41467-018-05083-x [Accessed 21 November 2024].
9. D. Risso, M. Cole, *scRNAseq: Collection of Public Single-Cell RNA-Seq Datasets* (2023).
10. G. Monaco, *et al.*, RNA-Seq Signatures Normalized by mRNA Abundance Allow Absolute Deconvolution of Human Immune Cell Types. *Cell Rep* **26**, 1627-1640.e7 (2019).
11. I. Korsunsky, *et al.*, *harmony: Fast, Sensitive, and Accurate Integration of Single Cell Data* (2023).
12. A. Regev, *et al.*, The Human Cell Atlas. *Elife* **6**, e27041 (2017).

13. M. D. Robinson, D. J. McCarthy, G. K. Smyth, edgeR: a Bioconductor package for differential expression analysis of digital gene expression data. *Bioinformatics* **26**, 139–140 (2010).

14. E. Y. Chen, *et al.*, Enrichr: interactive and collaborative HTML5 gene list enrichment analysis tool. *BMC Bioinformatics* **14**, 128 (2013).

15. R. M. Baldarelli, *et al.*, Mouse Genome Informatics: an integrated knowledgebase system for the laboratory mouse. *Genetics* **227**, iyae031 (2024).

16. K.-A. L. Cao, *et al.*, mixOmics: Omics Data Integration Project. (2016). Deposited 19 October 2016.

Supplementary figures

A

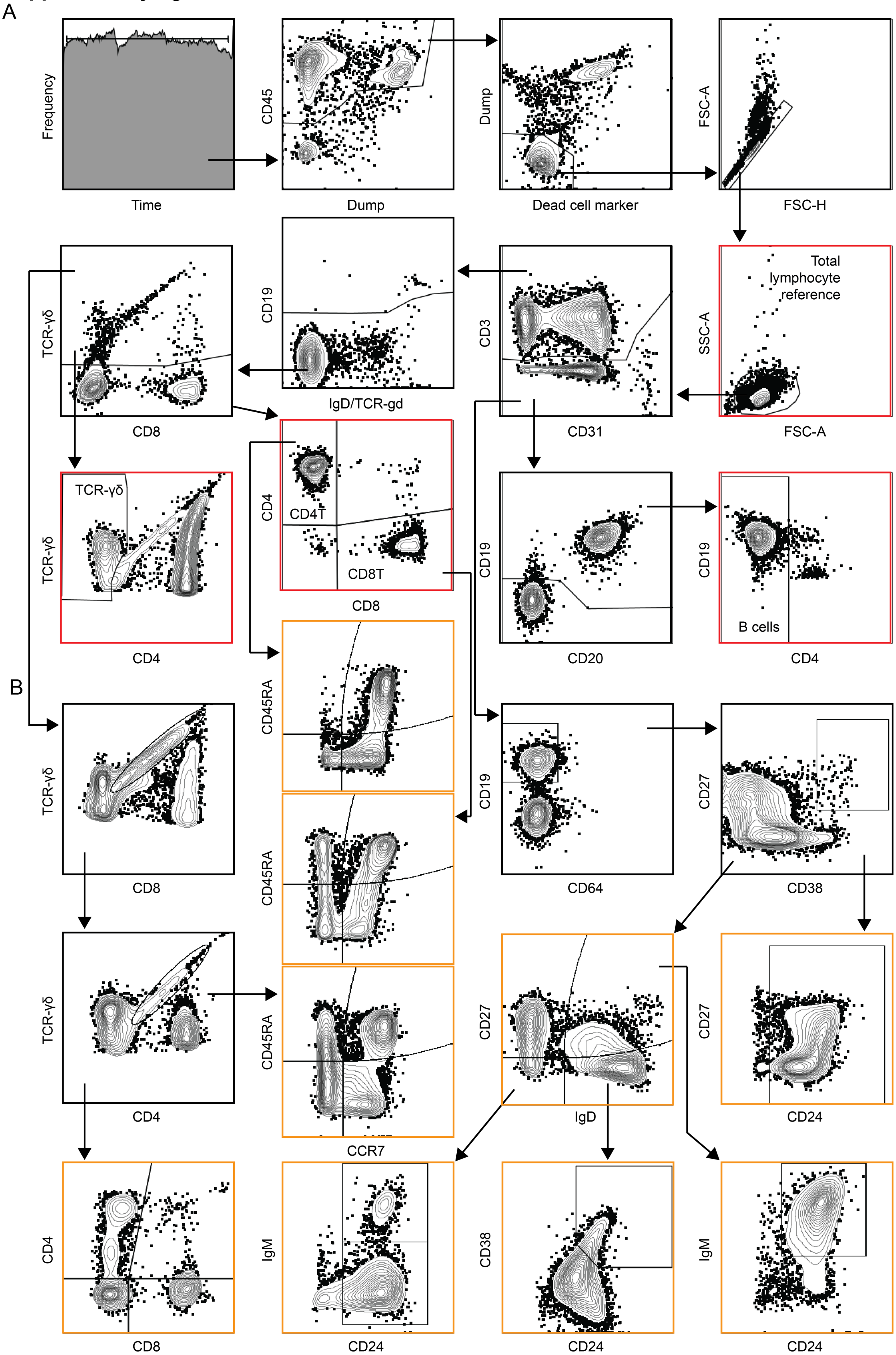

Supplementary figure 1. Gating strategy used for gating analysis and for Euclidean neighbor-based analysis for the B- and T-cell panel of the UK cohort. A: common gating strategy for gating

analysis (supplementary figure 5) and Euclidean neighbor clustering analysis. Red boxes indicate populations that were either picked out and used directly for down-stream analyses in the Euclidean neighbor-based analysis, or in the case of the total lymphocyte reference, was used as a control to calculate the relative abundance of the individual cell populations. B: gates used for gating analysis shown in supplementary figure 5. Orange boxes indicate that populations were drawn from this gate, both for direct frequency comparisons, but for the T-cell populations also for further identification of single-positive populations for a number of markers, described in supplementary figure 5A. All of these single-marker gates were created with histograms.

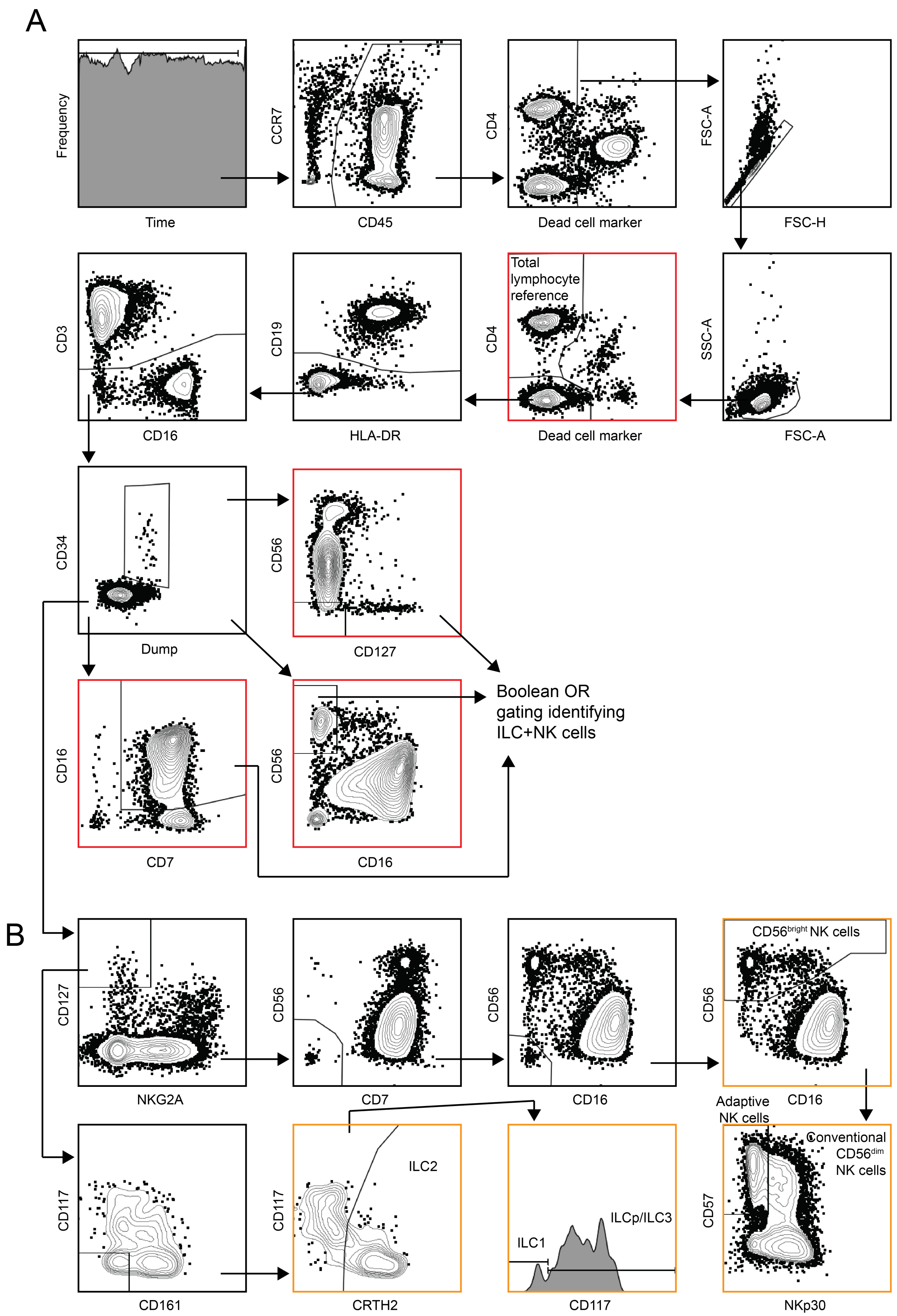

Supplementary figure 2. Gating strategy used for gating analysis and for Euclidean neighbor-based analysis for the ILC and NK cell panel of the UK cohort. A: common gating strategy for

gating analysis (supplementary figure 5) and Euclidean neighbor clustering analysis. Red boxes indicate populations that were either picked out and used directly for down-stream analyses in the Euclidean neighbor-based analysis, or in the case of the total lymphocyte reference, was used as a control to calculate the relative abundance of the individual cell populations. B: gates used for gating analysis shown in supplementary figure 5. Orange boxes indicate that populations were drawn from this gate, both for direct frequency comparisons, but for the T-cell populations also for further identification of single-positive populations for a number of markers, described in supplementary figure 5A. All of these single-marker gates were created with histograms.

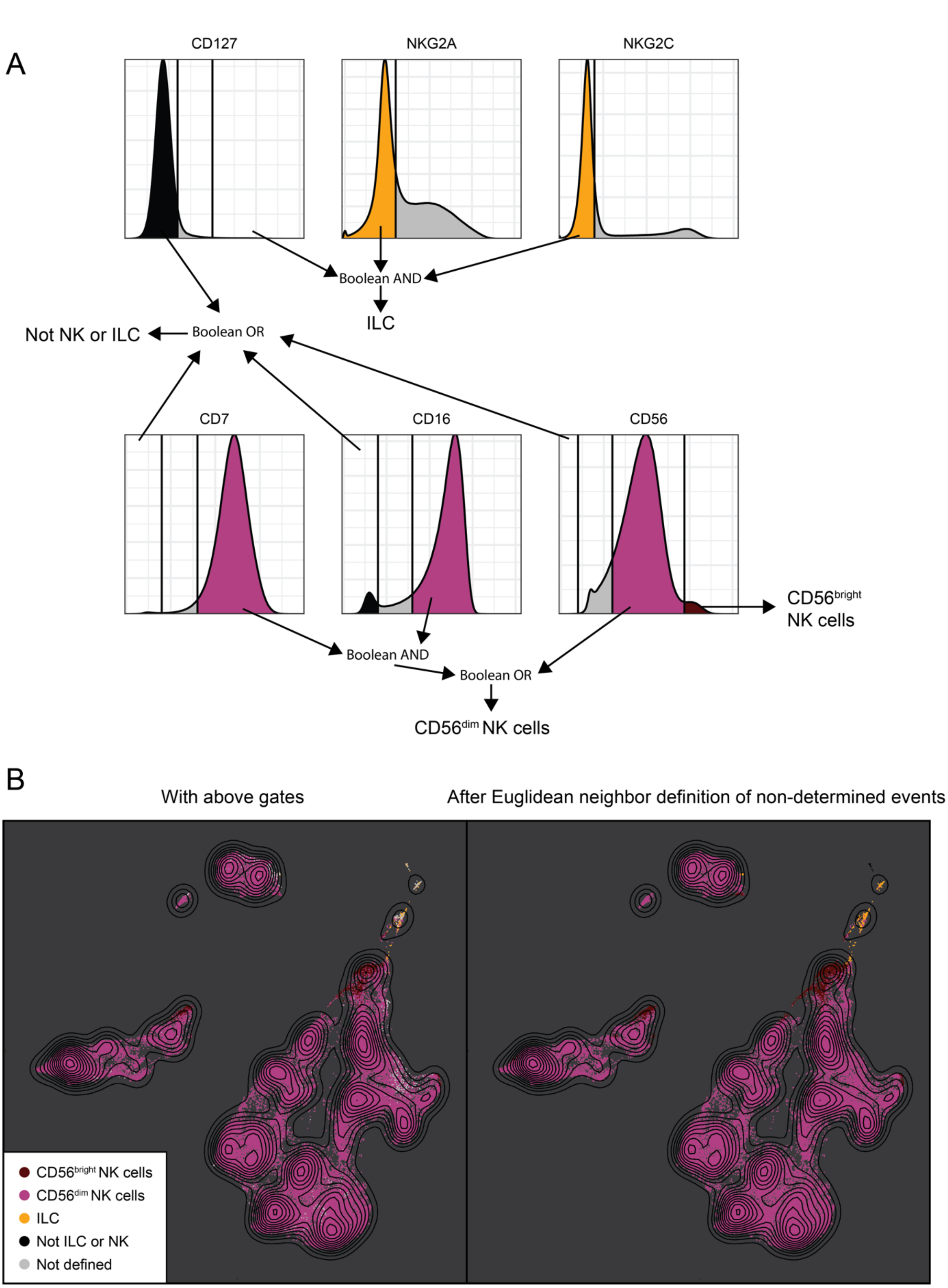

Supplementary figure 3. Euclidean-enhanced gating separating ILC and NK cells in the UK cohort. A: Histograms illustrating gates used to define the populations of interest. Four populations were defined: clearly CD127+NKG2A^{-}NKG2C^{-} cells were considered helper ILC, and within the non-hILC compartment, cells that were either clearly CD16 and CD7 positive or positive for CD56 were considered NK cells. Among the NK cells, CD56bright cells were dissociated from the rest. Within the cell population that did not clearly fulfill any of these criteria, a small population of cells clearly falling outside of the NK/ILC phenotype were defined as clearly negative for CD7, CD16, CD56 and CD127. B: The remaining populations, being outside any of these defined regions, were then associated to the

cell type of their Euclidean nearest neighbor in a space made up of NKp30, HLADR, CD183, CD161, CD16, CD34, CD56, CD8, CD127, CD27, CD57, NKG2A, CD2, NKG2C, CRTH2, CD7, CD117 and CD218a. After this procedure, the cells belonging to the non-ILC-nonNK cluster were excluded, and the CD56^{dim} and CD56^{bright} NK cells were re-merged. Small aberrant populations were also excluded from the hILC population at this stage, see supplemental methods section.

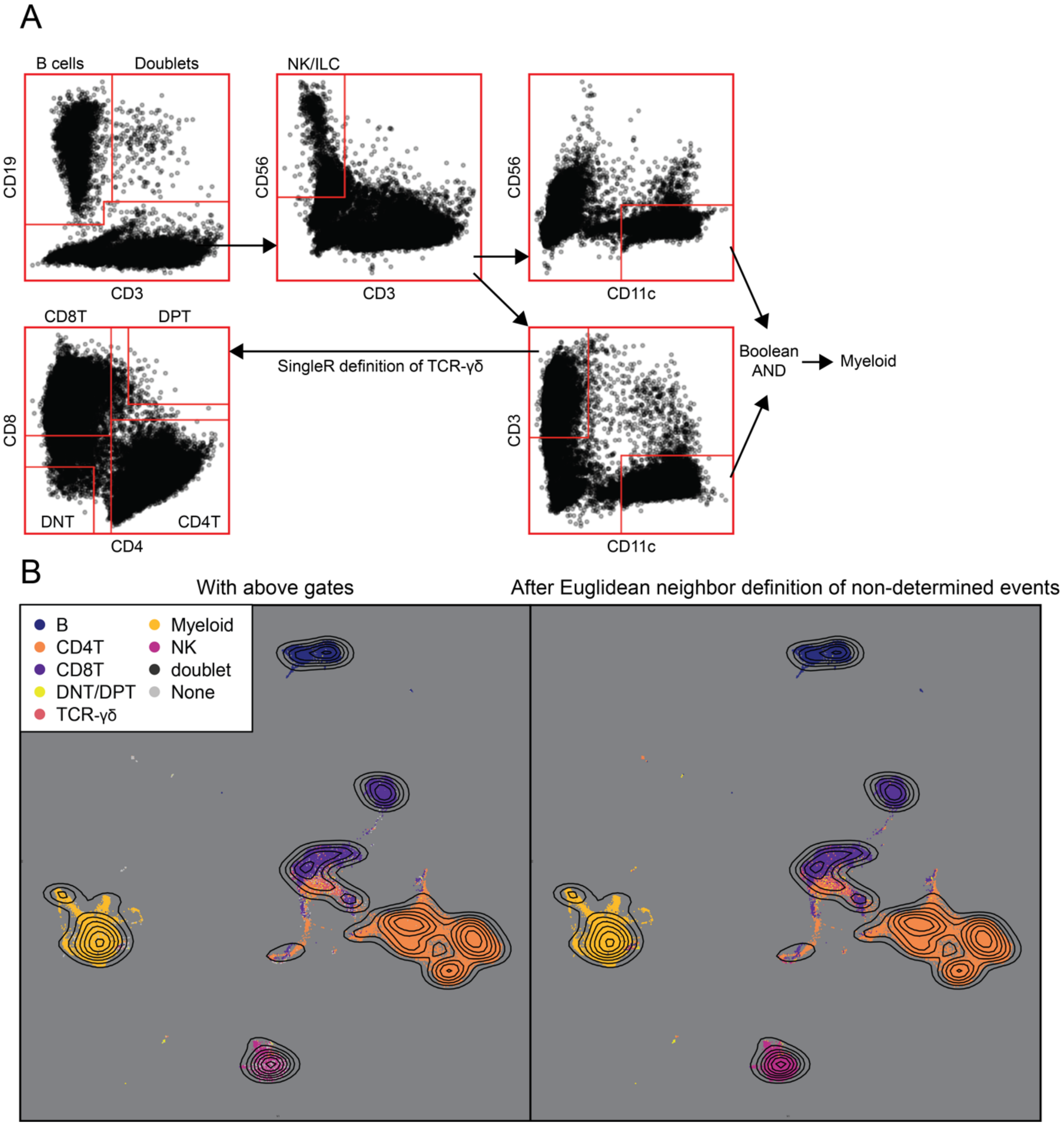

Supplementary figure 4. Euclidean-enhanced gating separating all cell subsets in the SE cohort. A: Histograms illustrating gates used to define the populations of interest. First, CD19-positive cells were defined as B-cells and CD3^{+}CD19^{+} cells were defined as B/T doublets. Then, clearly CD3^{-}CD56^{+} cells were defined as NK cells. This was followed by defining myeloid cells as CD11c^{+}CD3^{-}CC56^{-} and T-cells as CD3^{+}CD11c^{-}. The T-cells were then divided into CD4^{+}CD8^{-}, CD4^{-}CD8^{+}, CD4^{+}CD8^{+} (DPT) and CD4^{-}CD8^{-} DNT). B: The remaining populations, being outside any of these defined regions, were then associated to the cell type of their Euclidean nearest neighbor in a space made up of CD3, CD4, CD8, CD11c, CD16, CD19 and CD56 as well as the full transcriptome, integrated with totalVI.

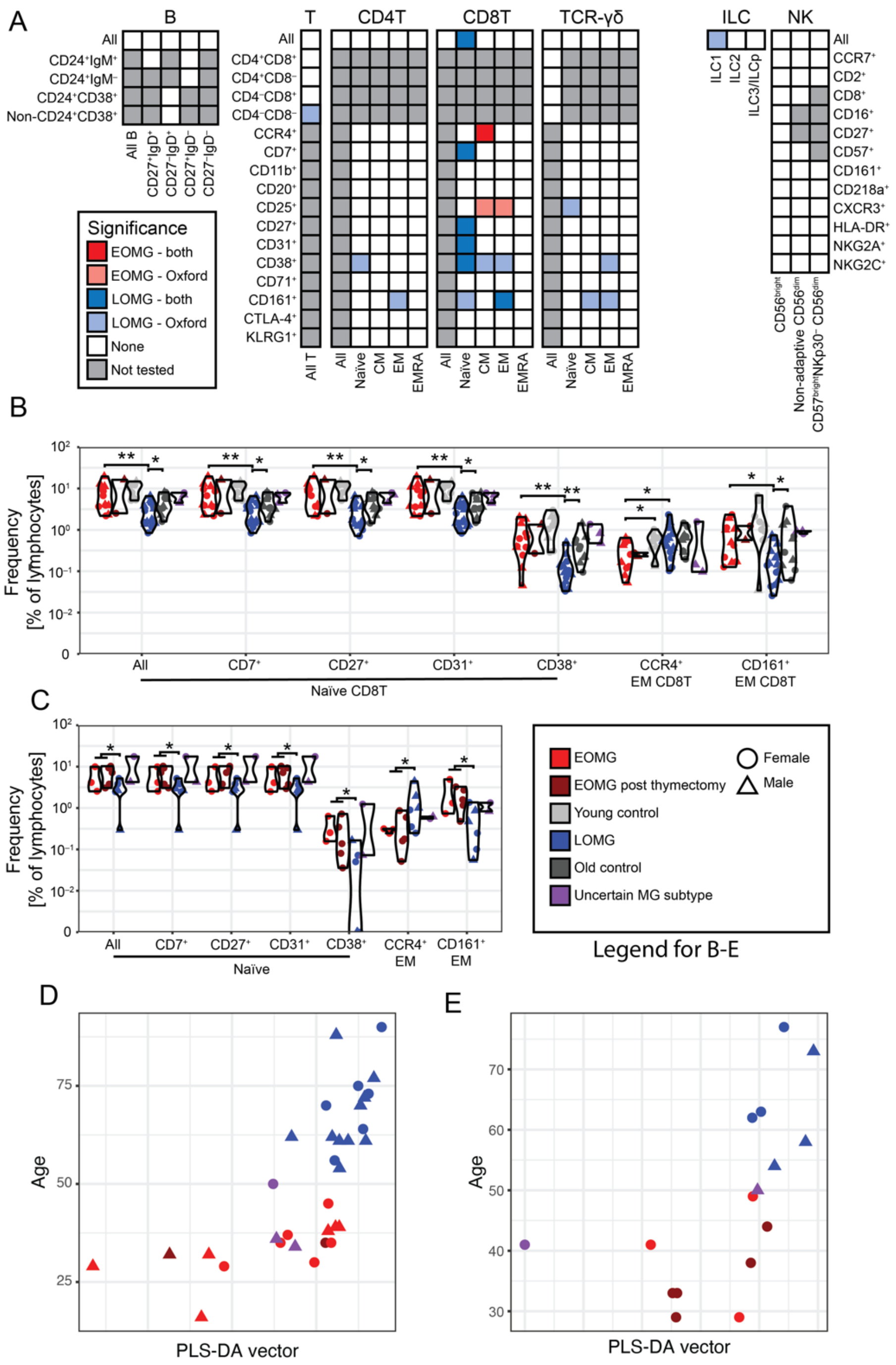

Supplementary figure 5. Gating analysis reveals differences within CD8 compartment insufficient for separation of the patient populations. In this analysis, conventional gating was first applied for the UK data (Supplementary figure 3 and 4), and gating in R was then performed on SE dataset on a per-cell type basis (the cell types were first separated through Euclidean-enhanced gating) for the clusters that showed signs of significance in the UK cohort. A: Overview of gated populations (there is a high degree of overlap between single-positive populations within each cell type). Light red and light blue indicate populations statistically separating EOMG and LOMG from both LOMG and controls in the UK data, respectively. Dark blue dots are LOMG populations that also differ in the SE data, thus considered confirmed discriminatory populations. B: distribution of frequencies of identified discriminatory populations between the patient and control populations in the UK. C: distributions for the same discriminatory populations but in the SE cohort. D,E: Partial least squares discriminant analysis, showing the best possible separation between the patient and control populations for the UK (D) and SE (E) cohorts, respectively.

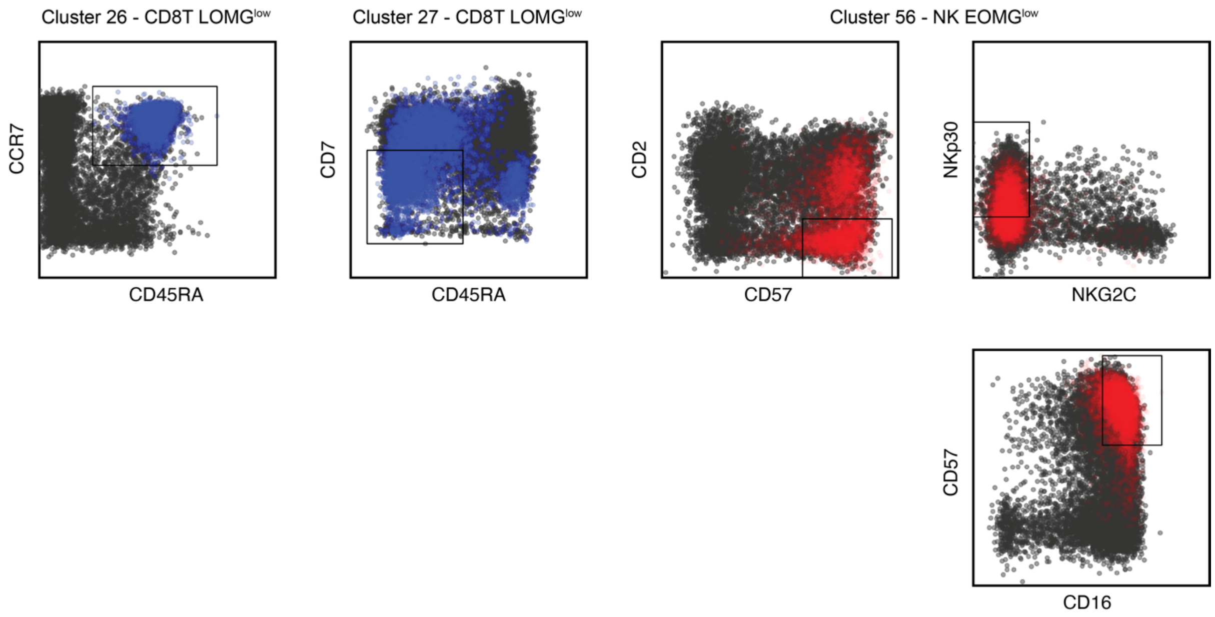

Supplementary figure 6. Conventional gates identifying discriminatory populations. Red and blue color indicates the population identified through the Euclidean neigbor smoothing principle. Setting gates in according to the pictures is however sufficient to identify a significantly different population for the UK data.

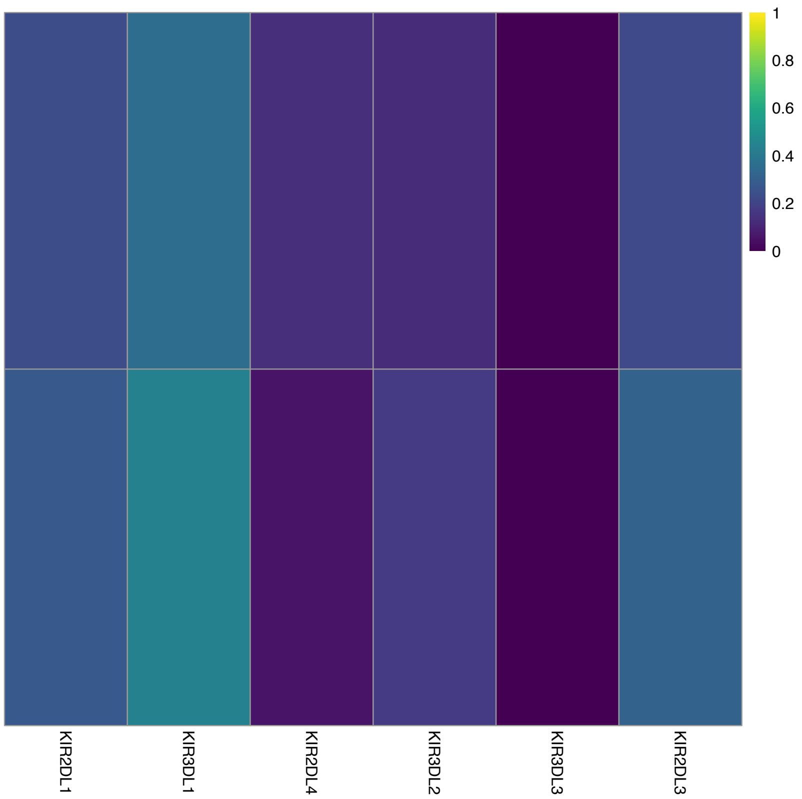

Supplementary figure 7. Inhibitory KIR gene expression. Heatmaps indicating fraction of cells expressing genes among the top differentially expressed proteins for each of the three significant populations. Row one shows all cells apart from the population of interest and row two the population of interest.

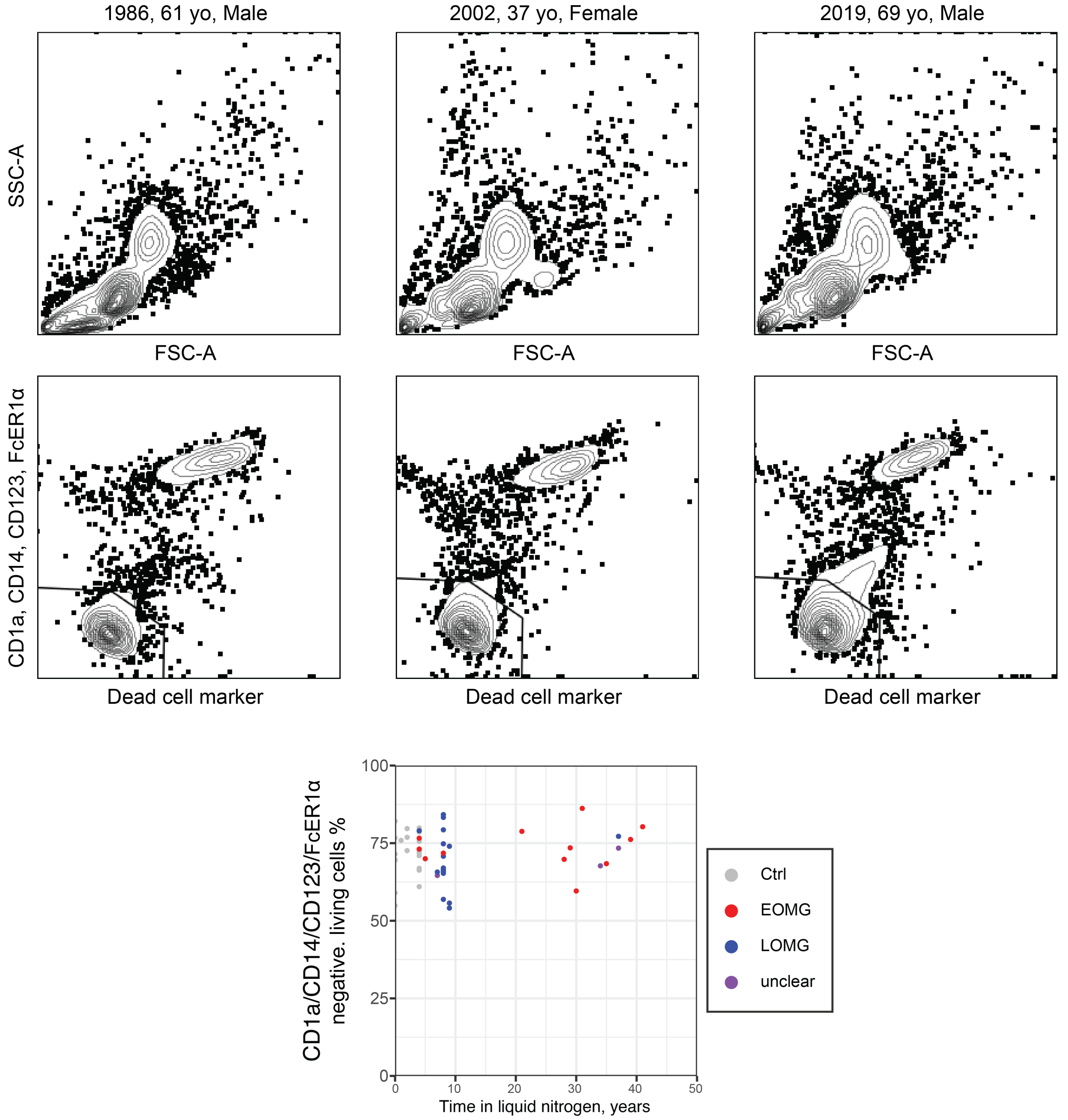

Supplementary figure 8. State of cells after decades in liquid nitrogen. A: Three representative examples of cells having spent 40, 20 or only a few years in liquid nitrogen stained with the B/T-cell panel. X-axis shows dead cell marker, whereas y-axis shows dump channel, mainly focused on the exclusion of myeloid populations. The emission spectrum of the dead cell marker (green) and the fluorophore in the dump channel (FITC) are very similar, and thus, there is considerable overlap between the dump-positive and the dead cell marker. However, the low number of cells in the area without clear myeloid staining patterns indicate a high viability rate in all samples. B: overview of the lack of correlation between the number of years in nitrogen tank and the frequency of cells in the live, dump-negative gate.

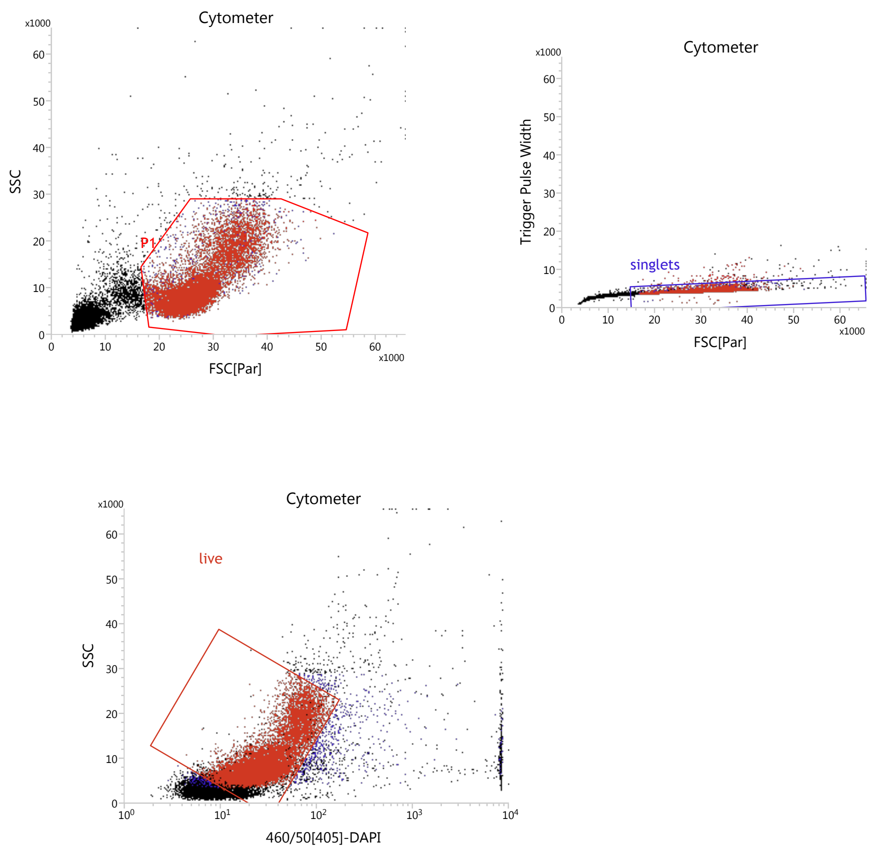

Statistics: Cytometer

Populations	Events	% Total	% Parent
All Events	20,000	100.00%	####
P1	13,419	67.10%	67.10%
singlets	13,315	66.57%	99.22%
live	12,686	63.43%	95.28%

Supplementary figure 9. Gates during sorting before multiomic analyses. Representative plots for one of the 16 samples from the SE cohort is shown. In accordance with the hierarchy, first, leukocytes were identified, then doublets were excluded and finally dead cells were removed.

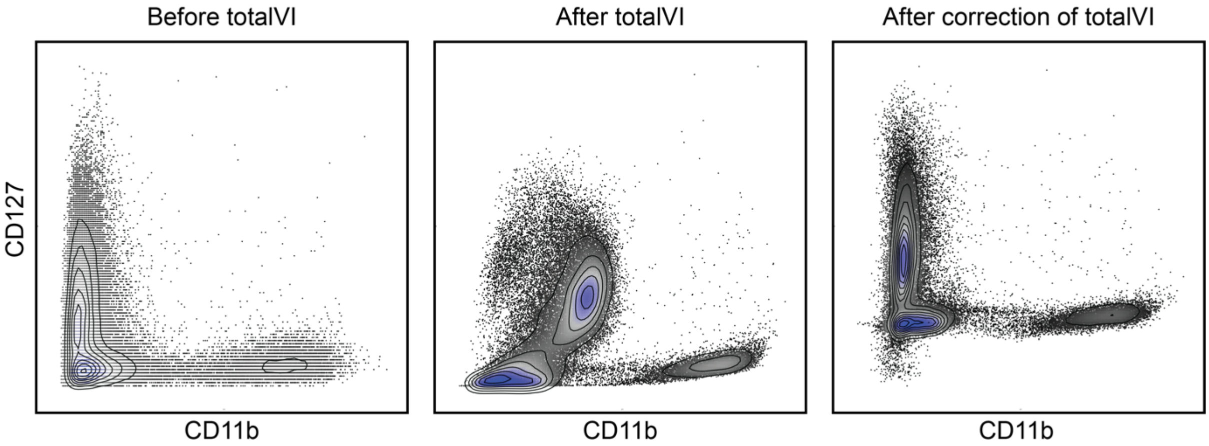

Supplementary figure 10. Introduction of artificial correlation between proteins by totalVI and mitigation of these correlations. Example of CD11b versus CD127, a combination with substantial totalVI impact. Left frame: CITE-seq data before totalVI analysis. Middle frame: introduction of artificial correlations. Right frame: the same data after linear model-based correction of the totalVI data.
